# A systematic review and meta-analysis of the prevalence of post-traumatic stress disorder (PTSD) in road traffic accident survivors

**DOI:** 10.34172/hpp.025.43651

**Published:** 2025-11-04

**Authors:** Kavous Shahsavarinia, Zahra Sabahi, Fateme Tahmasbi, Nooshin Milanchian, Haleh Farshi, Javad Mahmoudi, Mostafa Farahbakhsh, Hanieh Salehi-Pourmehr, Sakineh Hajebrahimi, Homayoun Sadeghi-Bazargani

**Affiliations:** ^1^Research Center for Evidence-Based Medicine, Iranian EBM Centre: A JBI Centre of Excellence, Faculty of Medicine, Tabriz University of Medical Sciences, Tabriz, Iran; ^2^Emergency and Trauma Care Research Center, Tabriz University of Medical Sciences, Tabriz, Iran; ^3^Department of Internal Medicine, Faculty of Medicine, Tabriz Medical Sciences, Islamic Azad University, Tabriz, Iran; ^4^Neurosciences Research Center, Tabriz University of Medical Sciences, Tabriz, Iran; ^5^Road Traffic Injury Research Center, Tabriz University of Medical Sciences, Tabriz, Iran

**Keywords:** Accidents traffic, Crashes traffic, Collision traffic, Motor vehicles, Post-traumatic neuroses, Stress disorders, Post-traumatic

## Abstract

**Background:**

This systematic review and meta-analysis aimed to evaluate the prevalence of post-traumatic stress disorder (PTSD) among road traffic accident (RTA) survivors, a demographic impacted by over 50 million disabilities globally each year.

**Methods:**

An initial systematic search was conducted in November 2021, with an updated search performed in October 2024. Relevant databases were comprehensively searched using keywords related to "traffic accidents," "road accidents," "motor vehicle accidents," "PTSD," and "Post-Traumatic Stress Disorder." This systematic review and meta-analysis were conducted in accordance with the PRISMA 2020 guidelines and the PICO framework established by Cochrane. The review included studies that involved RTA survivors diagnosed with PTSD, focusing on time frames from one to six months post-accident and utilizing DSM criteria. Eligible studies were reviewed for quality using the standardized critical appraisal instruments from the Joanna Briggs Institute (JBI) Checklist, by two independent reviewers. Meta-analysis was performed using Comprehensive Meta-Analysis statistical software and STATA16 to estimate overall prevalence rates and subgroup analyses to explore variations.

**Results:**

A comprehensive search across multiple databases identified 11,142 articles, of which 92 were reviewed, and 82 were included in the meta-analysis. The findings revealed an overall PTSD prevalence of 20.3% (95% confidence interval [CI]: 18.1%-22.8%; I^2^: 93.86%); 18.7% (95% CI: 16.0%-21.8%; I^2^: 93.47%) based on clinician-administered assessments and 22.8% (95% CI: 18.8%-27.3%; I^2^: 93.92%) from self-reported questionnaires. After removing outliers, the total prevalence was decreased to 18.1% (95% CI: 15.4%-21.0%; I^2^: 93.09%), in clinician-administered and 20.8% (95% CI: 17.5%-24.4%; I^2^: 91.51%) in self-reported questionnaires. Notably, the prevalence was 29.4% (95% CI: 22.4%-37.5%) one-month post-RTA, decreasing to 18.8% (95% CI: 14.8%-23.5%); *P*<0.001 at three months. Age did not significantly predict PTSD prevalence rates. The quality assessment of the studies included demonstrated moderate to high quality according to the Joanna Briggs Institute standards, ensuring the reliability of the findings. Geographic variability in PTSD prevalence was observed, with lower rates reported in Switzerland, Australia, Germany, and Japan, while higher rates were found in Spain, China, and Iran.

**Conclusion:**

This review highlights a significant PTSD prevalence of 20.3% among traffic accident survivors, emphasizing the need for early intervention and targeted mental health support to mitigate long-term psychological impacts and improve recovery outcomes in this vulnerable population. Screening and public awareness of disease symptoms are recommended.

## Introduction

 Even with worldwide and local initiatives aimed at preserving life on the roads, approximately 1.35 million people die each year due to road accidents.^1^ Road traffic accidents (RTAs) remain the third leading cause of disability, involving more than 50 million people injured worldwide annually. RTAs, as the most frequent accidental traumatic events, can happen to anyone, including children and teenagers. Evidence has shown that RTAs may increase a person’s risk of developing a wide range of psychiatric disorders, including post-traumatic stress disorder (PTSD), depression, and anxiety.^2-4^

 PTSD is a chronic and debilitating mental condition that may develop in response to catastrophic life events following exposure to an unintended traumatic event. Over the past few decades, there has been an increase in the number of people affected by traffic accidents worldwide. PTSD is primarily caused by motor vehicle accidents (MVAs) in the general population.^5^ In the United States, approximately six million motor vehicle accidents occur annually, causing over 2.5 million injuries. A study by the National Institute of Mental Health found that more than 39% of those who survive these accidents develop PTSD.^6^ Numerous studies on traumatic events have consistently demonstrated that PTSD can have long-term negative impacts on quality of life. The prevalence of PTSD varies widely between studies, ranging from 4.9% to 34.5%,^7-9^ which may be related to differences in the time interval between the trauma’s occurrence and the assessment of PTSD.

 The Diagnostic and Statistical Manual of Mental Disorders-V, fifth edition, outlines that PTSD is characterized by infiltration, avoidance, heightened arousal, and detrimental alterations in mood and cognition.^10,11^ Furthermore, PTSD can result in financial difficulties in addition to physical and psychological harm.^12^ Children and adolescents who have experienced RTA often suffer from diminished health-related quality of life due to early signs of PTSD.^13^ Additionally, individuals with PTSD are at an increased risk of metabolic syndrome and obesity.^14^ The medical costs for RTA survivors with PTSD are significantly higher than for those without PTSD. Accurate estimation of PTSD prevalence among RTA survivors helps health service providers provide prompt and effective intervention strategies.^15^

 Several factors are considered potential predictors of PTSD among survivors of MVAs. Previous studies indicate that female sex, depression, a history of RTA, peritraumatic dissociative experiences, an acute stress disorder (ASD) diagnosis, rumination, higher injury severity, and involvement in litigation or compensation following trauma are significant predictors of PTSD.^16-18^ Additionally, high levels of emotion (such as fear, helplessness, panic, guilt, or shame) during or right after a traumatic event, a lack of social support following a traumatic event, and previous psychological adjustment issues are all factors that increase the risk of developing PTSD.^17^ While injuries to the driver or passengers did not receive much support as predictive factors, the individual’s perceptions and responses to the accident, avoidance behavior, and suppressed thoughts about the accident significantly predicted PTSD.^16^

 A comprehensive understanding of PTSD prevalence is essential for the development of effective treatment strategies, informing policy decisions, and ultimately enhancing mental health outcomes for both individuals and communities. Two prior systematic reviews have been published to assess the prevalence of PTSD after RTA and to identify the predictors of PTSD in adult survivors of road traffic collisions.^15,19^ This systematic review and meta-analysis aim to investigate the prevalence of PTSD among survivors of RTA by updating previously published systematic reviews.

## Methods

 This systematic review and meta-analysis was conducted in accordance with the PRISMA 2020 guidelines, which provide a standardized framework for transparent and comprehensive reporting of systematic reviews and meta-analyses.^20^ The PRISMA checklist was followed to ensure that all critical elements of the review process were addressed, including the identification, screening, eligibility, and inclusion of studies.

###  Study Population

 Following the PICO framework established by Cochrane, we have previously articulated our intention to conduct a systematic review focusing on survivors of RTAs (Population) to investigate and synthesize the total prevalence (Outcome).

###  Inclusion/Exclusion Criteria 

 The following criteria were considered for eligibility: the sample consisted of RTA survivors diagnosed with PTSD 1 to 6 months after the accident (using different versions of DSM criteria or self-reported questionnaires). Studies were excluded if they were presented in non-English, were conference abstracts, were case studies or dissertations, were letters, or reviews. Additionally, while a month or more should pass before a PTSD assessment is conducted (no more than six months in line with DSM-IV criteria), studies on delayed PTSD and cases influenced by confounding variables, such as traumatic brain injury or post-traumatic amnesia, were excluded. No age restrictions were considered.

###  Information Sources

 An initial systematic search was conducted in November 2021, with an updated search performed in October 2024 in PubMed, Ovid, ProQuest, Scopus, Web of Science, Cochrane Library, and Google Scholar using the keywords Traffic Accident, Traffic Collision, Traffic Crash, Road accident, Motorcycle accident, Motorcar accident, Motor vehicle accident, PTSD, Moral Injury, and Post-traumatic Neuroses. The updated systematic search was conducted in October 2024 using the same search strategy and sources. Also, the reference lists of the studies were checked to retrieve any relevant publications. The full version of the search strategy is presented in Supplementary file 1.

###  Study Selection

 First, all identified citations were loaded into EndNote X20. After deleting duplicates, two independent reviewers screened titles and abstracts to assess the review’s inclusion criteria. Then, two independent professional reviewers (HS and ZS) evaluated the full text of the selected eligible studies in detail. If any did not meet the inclusion criteria, they were excluded. Any reviewer disagreements were resolved through discussion or by a third reviewer (HSB).

###  Data Extraction

 Utilizing the modified standard JBI data extraction tool, two reviewers (HS and ZS) independently determined the qualified papers and retrieved their data. Any disagreements were settled by consensus between the two reviewers or by conversing with the third reviewer (HSB). The data extraction table includes the study’s first author, publication year, the study’s nation, sample size, the timing of the PTSD assessment, the seriousness of the injury, the PTSD assessment tool, and prevalence. The PTSD diagnosis was determined through clinician assessments or self-reported questionnaires. The clinician-based evaluation contributed to the PTSD diagnosis, while the self-reported questionnaires indicated probable PTSD diagnosis.

###  Assessment of Methodological Quality

 According to standardized critical appraisal instruments from the Joanna Briggs Institute, eligible studies were critically appraised by two independent reviewers (HS and ZS) at the study level. Any disagreements were resolved by discussion or consultation with the third reviewer (HSB). Studies with a half or higher score in questions were included as high or moderate-quality studies (available at: https://jbi.global/critical-appraisal-tools).

###  Statistical Analysis

 All analyses were performed using registered copies of Comprehensive Meta-Analysis statistical software (version 3; Biostat, Englewood, NJ) and STATA16 (StataCorp, College Station, TX, USA). The study statistician extracted the data for the primary outcomes. The random effect model was employed because we might not have access to other unidentified, unregistered, or unpublished investigations. The between-study heterogeneity was assessed using statistics such as the Cochran Q test, Tau-squared, and I-squared. Significant results of the test and values higher than 75% for I-squared were considered substantial heterogeneity.^21^ Estimates were made for the effect sizes and 95% CIs. The publication bias was evaluated using funnel plots. To assess the bias, Egger’s^22^ and Begg’s^23^ as well as Duval and Tweedie’s trim and fill were performed. The age of the research population was used as the independent variable in the meta-regression analysis, which is typically necessary to identify the cause of heterogeneity.

 As needed, subgroup analysis based on the PTSD assessment tool by country, age groups (adults > 18 years, children < 18 years),^24^ and gender was conducted to identify the sources of heterogeneity.

## Results

###  Study Inclusion

**Figure 1:**
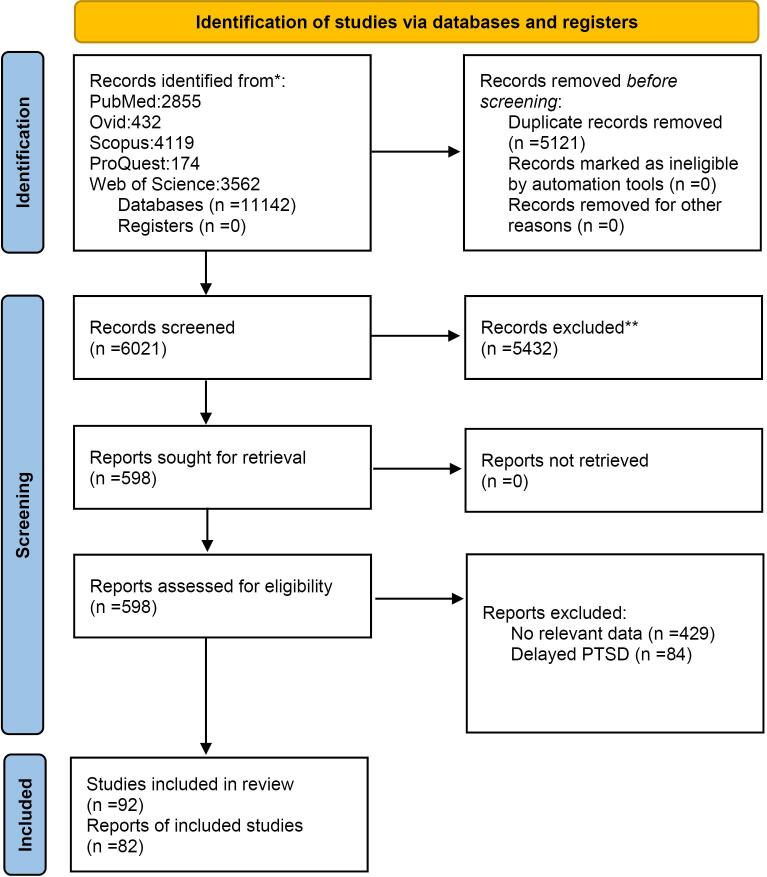
The PRISMA flow diagram shows the search and screening processes

 Our initial search retrieved 11142 articles from databases. After removing duplicates (n = 5121), reviewing the titles/abstracts, and reading the full text of eligible articles, 598 full texts of the articles were evaluated. Finally, 92 studies were systematically reviewed,^3,4,7,16,25-112^ and 82 studies (including 30 clinician-administered measures studies) were candidates for meta-analysis (Figure 1).

###  Characteristics of Included Studies

 The full details of the included studies are presented in Table 1. The prevalence of PTSD was evaluated in 82 studies.

**Table 1:** The characteristics of included studies

**First author (year)**	**Country**	**Sample size**	**Timepoint**	**Injury severity**	**Measure to assess PTSD and DSM version**	**Self-reported/ Clinician administered**	**PTSD prevalence**	**Mean age**
Alshardan (2020)^25^	Saudi Arabia	334	NM	NM	PCL-C (DSM-IV)	Self-reported	39.2	
Angerpointner (2020)^26^	Germany	36	6 weeks	Minor	IES-R	Self-reported	5.6	39.8 (16.0)
			3 months				2.8	
Bahari (2017)^27^	Malaysia	68	1 month	Minor, moderate, and major	Malay Post-Traumatic Stress Disorder Checklist Civilian version	Self-reported	7.4 (incidence)	66.09 (5.9)
Bedaso (2020)^28^	Ethiopia	416	Not less than one month	Hospitalized	PCL-S	Self-reported	15.4	
Berna (2012)^16^	France	155	6 months	Hospitalized	CAPS (DSM-IV)	Clinician administered	7.74	36.7 (16.4)
Bezabh (2018)^29^	Ethiopia	603			PCL-C	Self-reported	All emergency responders: 19.9 (ambulance nurses:11.5; firefighters:20.7)	
Blanchard (1995)^30^	USA	158	4 months	Sought medical attention	SCID (DSM-III-R)	Clinician administered	39.2	MVA victims: 35.4 (12.5) control: 37.7 (14.00)
Brand (2014)^31^	Germany	258	NM	Sought medical attention	Criterion A: Exposed to: death, threatened death, serious injury, and sexual violence (DSM-V)		0.78 (incidence)	
Bryant (2003)^32^	Australia	87	6 months	Hospitalized	CIDI-PTSD (DSM-III-R)	Clinician administered	22	Male: 29.95 (11.49) female:33.36 (13.28)
Bryant (2000)^33^	Australia	113	6 months	Hospitalized > 24 h	CIDI-PTSD (DSM-III-R)	Clinician administered	21	No-TBI: 33.70 (11.98)
Buckley (2004)^34^	USA	65	1 month	serious	SCID (DSM-IV)	Clinician administered	17	36.05 (15.04)
Chossegros (2011)^35^	France	541	6 months	Hospitalized	PCL (DSM-IV)	Self-reported	18	
Coronas (2011)^36^	Spain	119	1 month	Serious	SCID (DSM-III-R)	Clinician administered	45.4	38.3 (12.3)
		108	4 months				32.8	
Delahanty (2003)^37^	USA	59	1 month	Serious	SCID (DSM-IV)	Clinician administered	20	37.3 (17.7)
Doohan (2017)^38^ (bus)	Sweden	54	1-3 months	Minor, moderate, severe	TSQ		31% were high risk for PTSD	57
Ehlers (1998)^39^	UK	888	3 months	None, mild & moderate	PSS (DSM-IV)	Clinician administered	23.1	33.4 (13.1)
Ehring (2008)^40^	UK	141	6 months	Moderate to severe	SCID (DSM-IV)	Clinician administered	12.1	34.95 (10.60)
Fekadu (2019)^41^	Ethiopia	299	1 month	No major trauma	PCL-C	Self-reported	46.5	The median age was 31 with (IQR) of 25–42.
Fitzharris (2006)^42^	Australia	62 Males = 35 Females = 27	6-8 weeks	Hospitalized	PCL-C	Self-reported	Male: 2.9%, Female:7.4%	males: 35.3 (12.3), Females: 38.7 (12.3)
			6-8 months				Male: Nil, Female:7.4%	
Flesher (2001)^43^	USA	70	1 month	Hospitalized	SCID (DSM-IV)	Clinician administered	17	33.2 (14.6)
Fredman (2017)^44^	USA	114	4 weeks	Severe	PCL-C	Self-reported	42.1	38.14 (12.52)
			16 weeks				24.3	
Frommberger (1998)^45^	Germany	152	6 months	Hospitalized minimum of bone fracture	IES, PSS (DSM-III-R)	Self-reported	18.4	
Fuglsang (2004)^46^	Denmark	90	6–8 months	Attended ED	PDS (DSM-IV)	Self-reported	17	33.99 (11.3)
Fullerton (2001)^47^	USA	122	1 month	Serious	SCID (DSM-III-R)	Clinician administered	34.4	35.6 (13.1)
Gabert-Quillen (2012)^48^	USA	201	6 months	hospitalized	CAPS	Clinician administered	7	39.6 (15.7)
Hamanaka (2006)^4^	Japan	82	6 months	Serious	SCID (DSM-IV)	Clinician administered	8.5	
Harvey (1998)^49^	Australia	71	6 months	Hospitalized > 24 h	CIDI-PTSD (DSM-III-R)	Clinician administered	25.4	33.29 (12.00)
Holeva (2001)^50^	UK	265	4–6 months	Serious	Penn Inventory (DSM edition not specified)	Self-reported	23	
Hu (2018)^51^	China	70	6 months	mild	CAPS (DSM-IV)	Clinician administered	41.4	
Irish (2011)^52^	USA	196	6 weeks	Hospitalized	CAPS (DSM-IV)	Clinician administered	10.36	38.4 (14.7)
			6 months	Mild, moderate & severe			7.14	
Iteke (2011)^53^	Nigeria	150	1-12 months		PTSD module of the Mini International Neuropsychiatric Interview (M.I.N.I)	Clinician administered	26.7	RTA:31.61 ± 9.18, control 1: 32.14 ± 8.85, control 2: 33.01 ± 8.95
Jeavons (2000)^54^	Australia	72	3 months	Attended to hospital	PTSD-I (DSM-III-R)	Clinician administered	8.3	31.8 (12.78)
		62	6 months				8	
Jones (2005)^55^	UK	131	6 weeks	Severe	PSS (DSM-IV)	Clinician administered	Non-TBI: 27.4 TBI: 30.4	36.75 (12.77)
			3 months				Non-TBI: 18 TBI: 17.2	
Kassam-Adams (2009)^56^	USA	251 parents of children with RTA	6 months	Hospitalized	PTSD Checklist		8	
Khodadadi-Hassankiadeh (2017)^57^	Iran	528	6 weeks-6 months	Attended to hospital	PSS	Clinician administered	30.49	33.59 (13.29)
Kobayashi (2019)^58^	USA	280 (120 women, 160 men)	6 weeks	Admitted to level-1 trauma centers	CAPS (DSM-IV)	Clinician administered	Women: 19.2 Men: 8.1	women:39.93 (15.29), men: 37.41 (15.00)
		217 (99 women, 118 men)	6 months				Women: 14.1 Men: 2.5	
Kovacevic (2021)^59^	Balkans	200	1 month	Mild, moderate, serious, severe, critical	PCL-C	Self-reported	35.5	
			6 months				20.5	
Kuhn (2006)^60^	Germany	58	6 months	Moderate to Severe	SCID (DSM-IV German version)	Clinician administered	6	38.6 (13.6)
Kupchik (2007)^61^	Israel	60	3 months	Outpatient clinic	CAPS-2, SCID-I/P	Clinician administered	50	PTSD: 44.6 (11.1) non-PTSD: 45.4 (13.2)
Li (2021)^62^	China	206	4–12 months	Mild, severe, critically severe	PCL-S	Self-reported	24.8	39.8 (12.5)
Matsuoka (2008)^63^	Japan	100	4–6 weeks	Severe	CAPS (DSM-IV)	Clinician administered	8	37.0 (16.1)
Mayou (1993)^64^	UK	174	3 months	Minor & Major	Diagnostic criteria for PTSD (DSM-III-R)	Clinician administered	8	
Mayou (1997)^65^	UK	111	3 months	Attended ED		Clinician administered	10	
McFarlane (1997)^66^	Australia	26	6 months	Hospitalized	CAPS (DSM-IV)	Clinician administered	26.9	
Naim (2014)^67^	Israel	415	3 months	Minor, admitted to ED	CAPS, PCL, CADSS	Clinician administered	6.75	
Nightingale (2000)^68^	UK	60	6 weeks		PDS (DSM-IV)	Self-reported	30.8	T1:33.3 (10.5), T2:34.8 (10.8)
Ning (2017)^69^	China	166	3 months		PCL-C	Self-reported	15	38.75 (1.13)
Nishi (2013)^70^	Japan	106	6 months	Admitted to ICU	CAPS	Clinician administered	7.5	38.3 (16.0)
Ongecha-Owuor (2004)^71^	Africa	264	1 month	Serious	SPI (DSM-IV)	Clinician administered	13.3	
Papadakaki (2017)^72^	Greece, Germany and Italy	initial:120 (Greece = 41, Germany = 3, Italy = 40), 12 months: 93	6, 12 months	Admitted to ICU	IES-R	Self-reported	PTS: Baseline: 43.5% 6 months: 39.6% 12 months: 21.1%	41.8 (16.7)
Prakasam (2013)^73^	India	86	6 months	Moderate, major	IES-R	Self-reported	23.3	
Pires (2013)^74^	Portugal	124	4 months	serious	RTES		58.90	
Ryb (2009)^75^	USA	367	6 months	Hospitalized	Diagnostic criteria for PTSD (DSM-IV)	Clinician administered	27.5	
Saberi (2013)^76^	Iran	385	NM		PCL-C) Persian version(	Self-reported	19.2	35.45 ± 9.04
Shaikh (2012)^77^	France	21	2 months	hospitalized	CAPS	Clinician administered	33.3	At 2 months PTSD + : 23, PTSD - : 26 at 6 months PTSD + : 29, PTSD - : 20
		18	6 months				38.9	
Smith (2007)^3^	UK	39	4 months	Minor (out-patients)	SRS-PTSD (DSM-III-R), IES	Self-reported	12.8	
Suliman (2014)^78^	South Africa	Initial:131, 3 months: 104, 6 months: 101	3 months, 6 months	Minor, major	CAPS	Clinician administered	baseline: 22.9% 3 months: 19.6% 6 months: 12.2%	PTSD: 34.75 (11.54) no PTSD: 33.71 (11.16)
Sun (2013)^79^	China	62	6 months		CAPS	Clinician administered	33.9	Trauma-exposed victims with PTSD (N = 21): 40.86 ± 12.26 Trauma-exposed victims without PTSD (N = 17): 35.64 ± 11.91 Follow-up of trauma exposed victims with PTSD (N = 11): 42.09 ± 12.79 Healthy control: 40.23 ± 12.54
Ursano (1999)^80^	USA	122	1 month	Serious	SCID (DSM-III-R)	Clinician administered	34.4	35.6 (13.1) MVA: 35.59 (13.06), control 37.16 (13.09)
		99	3 months				25.2	
		99	6 months				18.2	
Vaiva (2003)^81^	France	123	2 months	Hospitalized	CAPS (DSM-IV)	Clinician administered	51	
Wang (2005)^82^	Taiwan	64	1 week	Hospitalized	PTSD-RI (DSM-III-R)	Self-reported	87.5	33 (11.77)
			6 weeks				82.8	
Yasan (2009)^83^	Turkey	84	3 months	Attended ED	CAPS (DSM-IV)	Clinician administered	29.8	
		78	6 months				23.1	
Yohannes (2018)^84^	Ethiopia	492	1 month		PCL-S	Self-reported	22.8	30.12 (7.02)
**Children **								
Bryant (2004)^90^	UK	86	3 months	Minor, or hospitalized	Post-Traumatic Stress Disorder Reaction Index (RI) (DSM-IV)	Self-reported	25	12.27 (2.86)
			6 months				18	
DI Gallo (1997)^91^	Scotland				PTSD-RI; IES	Self-reported		10.2 (3.6)
		49	12-15 weeks				Mild: 35; moderate: 8; severe: 6	
Gillies (2003)^92^	Scotland	158	baseline	Attended ED	CPTS-RI, CAPS-C	Clinician administered	Mild: 48 Moderate: 18 Sever: 0	
			2-16 days				Mild: 33 Moderate: 7 Sever: 7	
			12-15 weeks				Mild: 44 Moderate: 22 Sever: 7	
Jones-Alexander (2005)^93^	Albany	21			CPTSDI, PCL-C		38.1	12.7
Landolt (2005)^94^ (PTSS)		68	4–6 weeks	Hospitalized	Child PTSD Reaction Index (RI)(DSM-IV-TR); PDS for parents		16.2; mothers (20%); fathers (11.3%)	Children: 9.82 (2.55)
Allenou (2010)^95^	France	Mothers: 72 Fathers: 28	5 weeks	NM	PCL-S	Self-reported	18.1 in mothers 3.6 in fathers	Age of fathers: 40.9 (5.3); Age of mothers 41.7 (6.2)
Meiser-Stedman (2009)^96^	UK	28	2-4 weeks	Attended ED	RIES-C; CPTC	Self-reported	21.4	13.2 (1.9)
			6 months				10.7	
Mirza (1998)^97^	UK	119	6 months	Attended ED	FRI and the PTSD symptom checklist (DSM-IV).	Clinician administered	Severe: 12 moderate:1.7 mild:3.4	13.61 (2.44 years)
Pervanidou (2007)^98^	Greece	56	1 month	minor, moderate and serious	K-SADS; CPTS-RI	Clinician administered	41.1 Boys: 32 Girls: 7	children: 10.70 (2.46), control: 10.49 2.59
		48	6 months				18.8 Boys: 16 Girls: 2	
Salter (2004)^99^	UK	67	few weeks	Admitted to hospital	CAPS-C	Clinician administered	37	15 (3)
Schäfer (2006)^7^	Germany	72	1 week	Attended ED	Impact of Event Scale – Revised (IES-R)	Self-reported	11	13.6(3.3)
		69	3 months				25	
Stallard (2001)^100^	USA	97	6 weeks	Attended ED	CAPS-C	Clinician administered	37.1	14.62 (3.16)
Stallard (2004)^101^	UK	158	4 weeks	Attended ED	CAPS-C	Clinician administered	29.1	14.85 (3.11)
Williams (2015)^102^	USA	3604	6 months	Serious	NWS	Clinician administered	7.4	14.63 (1.66)
Wu (2016)^103^	China	537	3 months	Admitted to the hospital	CAPS-CA	Clinician administered	24.77	6.8 ± 0.9
Zehnder (2010)^104^	Switzerland	50	2 months	Hospitalized	CAPS-CA	Clinician administered	7.1	7-16 years
		50	6 months				4	
Maeda (2009)^105^ (ship)	Japan	Adolescent: 9 Adult: 17	2 months		CAPS	Clinician administered	Adolescent: 77.8 Adult: 12	student: 17.0 (0.0), crew: 45.9 (11.6)
Giannopoulou (2021)^106^	Greece	168	2 months		Children’s Revised Impact of Events Scale (CRIES-13)	Self-reported	78	14.5 (1.3)
Ziobrowski (2021)^107^	USA	1003	3 months		CAPS-DSM-IV PTSD	Clinician administered	26.60	34.5 [24-43]
Kessler (2021) ^108^	USA	666	2 months		PCL-5	Self-reported	39.00	-
Joormann (2022) ^109^	USA	1306	3 months		PCL-5	Self-reported	20	-
Arora (2021)^110^	India	250	1-12 months		PCL-5	Self-reported	32.40	31-45
Yrondi (2022)^111^	France	125	5 weeks, 6 months		PCL-5	Self-reported	5 weeks: 13.6, 6 months: 10.3	40.83 (5.21)
Neylan (2021)^112^	USA	666	2 months		PCL-5	Self-reported	42	-
**Airplane**								
Lesaca (1996)^85^	USA	Trauma counseling: 21 no trauma counseling:20	4 weeks	NM	DSM-IV	Clinician administered	48 10	
		Trauma counseling: 21 no trauma counseling: 20	8 weeks				24 0	
		No trauma counseling: 20	12 weeks				14 25	
**Train accident**								
Engelhard (2002)^86^	Belgium	Directly exposed: 29	3 weeks	Serious	PSS	Clinician administered	28	53 (17)
			3.5 months				17	
Kim (2013)^87^ (subway drivers)	South korea	826	NM	NM	K-CIDI 2.1	Clinician administered	1.5	
Lemos (2018)^88^	Portugal	216	Baseline		PCL-C (Portuguese version)	Self-reported	8.3	
			Less than a month				37.5	
			6 months				10.2	
Mehnert (2012)^89^	Germany	71	1 month		Posttraumatic Diagnostic Scale (PDS) — German version	Self-reported	moderate PTS: 28%, moderate to severe PTS:42%, severe PTS: 11%	48 (7.8)
		49	6 months				moderate PTS: 29%, moderate to severe PTS: 29%, severe PTSD: 8%	

 The research encompassed a diverse range of countries, including Saudi Arabia,^25^ Albania,^93^ Australia,^32,42,49,54,66^ Belgium,^86^ China,^51,62,69,79,103^ Scotland,^92^ Denmark,^46^ Ethiopia, ^28,29,41,84^ France,^16,35,77,81,95,111^ Germany,^7,26,31,45,60^ Greece,^98,106^ India,^73,110^ Iran,^57,76^ Taiwan,^82^ Israel,^61,67^ Japan,^4,63,70,105^ Malaysia,^27^ Nigeria,^53^ Portugal,^74^ South Korea,^87^ South Africa,^71,78^ Spain,^36^ Sweden,^38^ Switzerland,^104^ Turkey,^83^ the United Kingdom,^3,39,40,50,55,64,65,68,90,96,97,99,101^ Balkan,^59^ Greece, Germany, and Italy,^72^ and the United States.^30,34,37,43,44,47,48,52,56,58,75,80,100,102,107,108,109,112^ Some studies reported the prevalence of PTSD in children.^7,85,90-92,93-106^

 The studies varied in geographical distribution, with 39 conducted in Europe, 20 in Asia, 19 in North America, 7 in Oceania, and 7 in Africa. Sample sizes were notably heterogeneous, ranging from a minimum of 21^77,93^ to a maximum of 3,604 participants,^102^ and assessment durations spanned from several days to six months. The severity of injuries was documented through qualitative measures.

 Diagnostic assessments predominantly utilized standardized instruments such as the Clinician-Administered PTSD Scale (CAPS) aligned with DSM-IV criteria, the PTSD Checklist-Civilian version (PCL-C), the Structured Clinical Interview for DSM (SCID) as per DSM-III-R or DSM-IV standards, the Impact of Event Scale-Revised (IES-R), the PCL-S, the Composite International Diagnostic Interview for PTSD (CIDI-PTSD), the PTSD Diagnostic Scale (PDS), the Perceived Stress Scale (PSS), the Penn Inventory, and the PTSD of the Mini International Neuropsychiatric Interview (M.I.N.I). Although less commonly employed, additional evaluation methods are comprehensively outlined in Table 1.

 Among the studies reviewed, PTSD diagnoses were self-reported in 35 cases and clinician-administered in 50 cases. The reported prevalence of PTSD varied widely, falling within the range of 2.9% to 77.8%. Furthermore, the mean age of participants spanned from 23 years to 66.09 years, with a standard deviation of 5.9 years. The time point to measure PTSD was between 1 to 6 months after RTAs in survivors.

 We analyzed the frequency of PTSD according to the clinician-administered or self-reported questionnaire in the included studies at different time points. According to the results of clinician-administered assessment, the prevalence of PTSD varied between the minimum percent of 13.3% in Ongecha-Owuor et al^71^ to 48% at the Lesaca et al^85^ study one month after RTA. At six weeks after RTA, the prevalence of PTSD varied between 10.4% in the Irish et al study^52^ to 37.1% in the Stallard study. The minimum and maximum rate was 7.1%^104^ to 51%^81^ at two months post-injury, respectively. At three months post-injury, this rate was 6.8%,^67^ and 50%,^61^ and at 6 months 1.7%,^97^ and 38.9%,^77^ respectively.

 In terms of self-administered questionnaires, the prevalence rate of PTSD was low in the study of Allenou et al from France (3.6%)^95^ at one month post-injury, while Fekadu et al^41^ reported the highest rate (46.5%). At 6 weeks post-injury, Angerpointner et al^26^ reported 5.6% of PTSD, and Wang et al^82^ reported 82.8% of cases with this disorder. At three months, the Angerpointner et al^26^ study found that only 2.8% of cases had PTSD, while Bryant et al^90^ from the UK, and Schäfer et al^7^ from Germany reported 25% of its prevalence.

 The minimum rate of PTSD after RTA was observed in the Mehnert et al^89^ study (8.0%), and the maximum rate was in the Papadakaki et al^72^ study (39.6%).

####  Clinician-Administered Measures 

**Figure 2:**
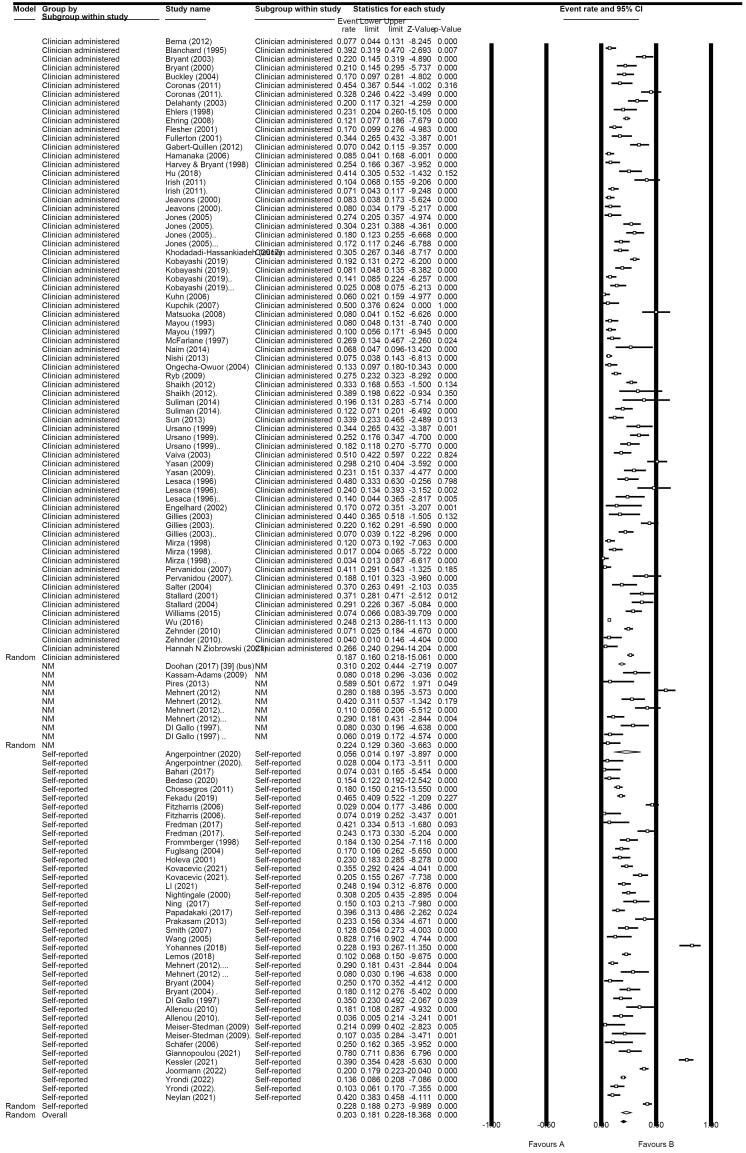
The forest plot for the prevalence of PTSD based on Clinician-administered and self- reported groups

 We calculated the total prevalence of PTSD according to the clinician-administered measures, which was 18.7% (95% CI: 16.0%-21.8%; I^2^: 93.47%) (Figure 2).

 After removing outliers, the total prevalence decreased to 18.1% (95% CI: 15.4%-21.0%; I^2^: 93.09%) in clinician-administered.

**Figure 3:**
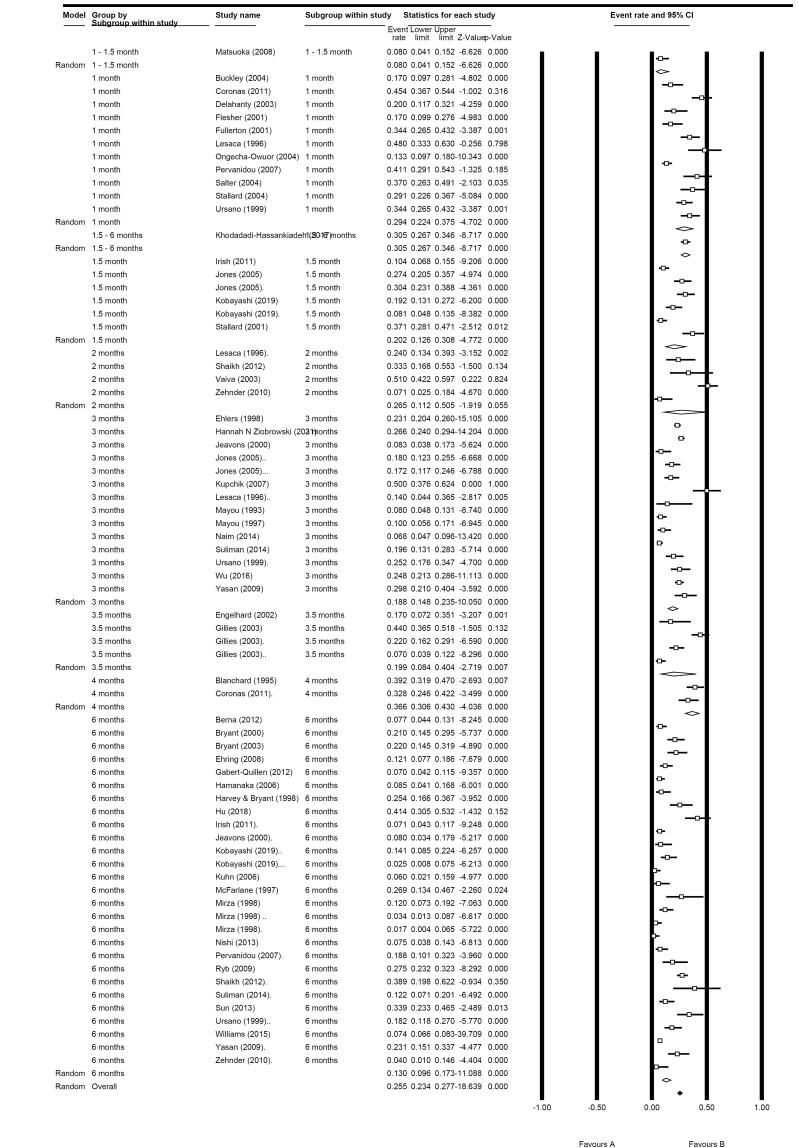
The forest plot for the prevalence of PTSD at different time points within Clinician-administered group

 In subgroup analysis based on the time points in clinician-administered measures-related studies, the results revealed that 1 month after RTA, the prevalence of PTSD was 29.4% (95% CI: 22.4%-37.5%; I^2^: 85.97%) among 11 eligible studies; in 3 months following RTA, this rate was 18.8% (95% CI: 14.8%-23.5%; I^2^: 89.83%) in 13 included studies, and at six months was 13.0% (95% CI: 9.6%-17.3%; I^2^: 95.81%) in the 24 included studies. At the other time point, consisting of 2 months following RTA, this rate was 26.5% (95% CI: 11.2%-50.5%; I^2^: 88.52%) in 4 eligible studies, and at 4 months was 36.6% (95% CI: 30.6%-43.0%; I^2^: 11.49%) in two eligible studies (Figure 3).

**Figure 4:**
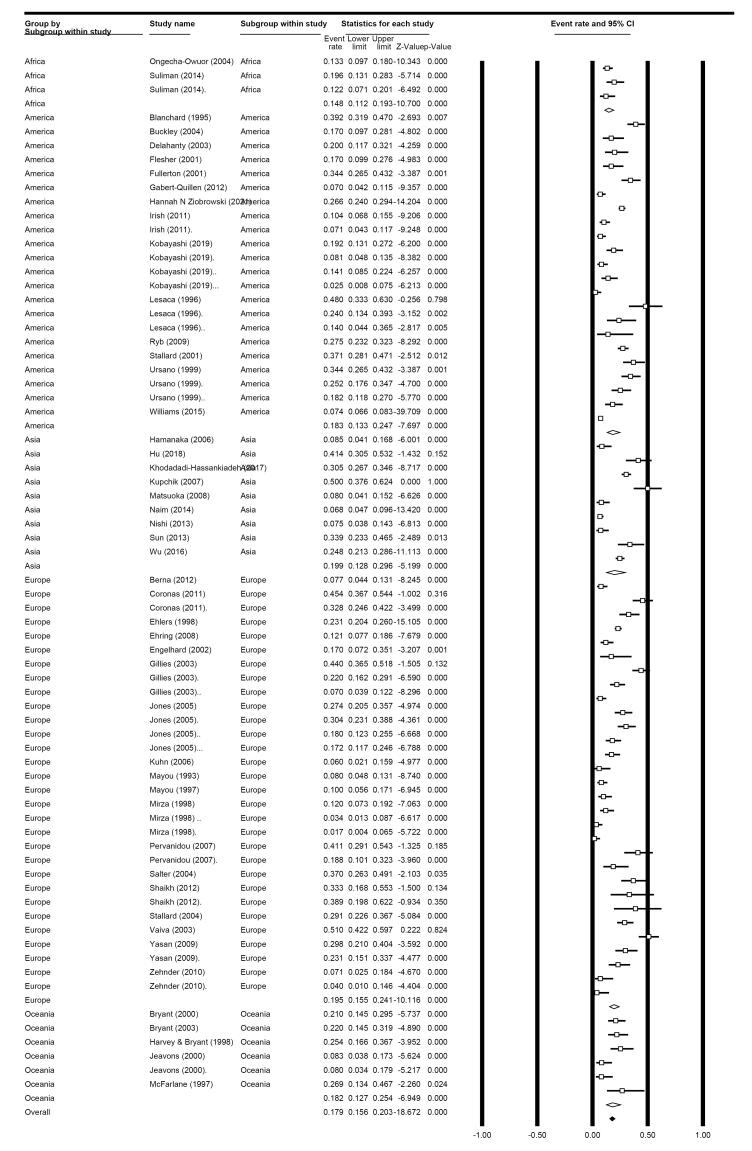
The forest plot for the prevalence of PTSD in different WHO regions within Clinician-administered group

 In subgroup analysis based on continents in clinician-administered measures-related studies, the prevalence in American areas was 18.3% (95% CI: 13.3%-24.7%) among 14 studies; in European regions, 19.5% (95% CI: 15.5%-24.1%) in 19 eligible studies, 18.2% (95% CI: 12.7%-25.4%) among six related studies in Western Pacific regions, 19.9% in Asia (95% CI: 12.8%-29.6%) in nine eligible studies, and 14.8% (95% CI: 11.12%-19.3%) in 3 African studies (Figure 4).

**Figure 5:**
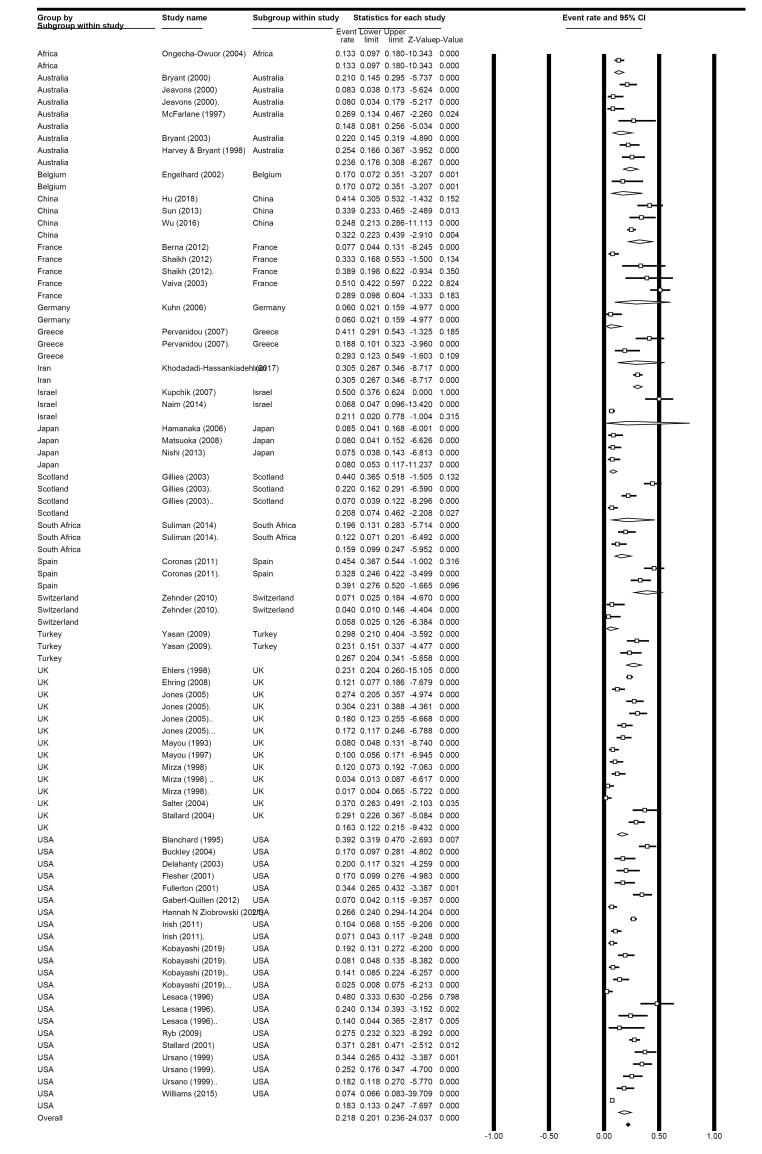
The forest plot for the prevalence of PTSD in different countries within Clinician-administered group

**Figure 6:**
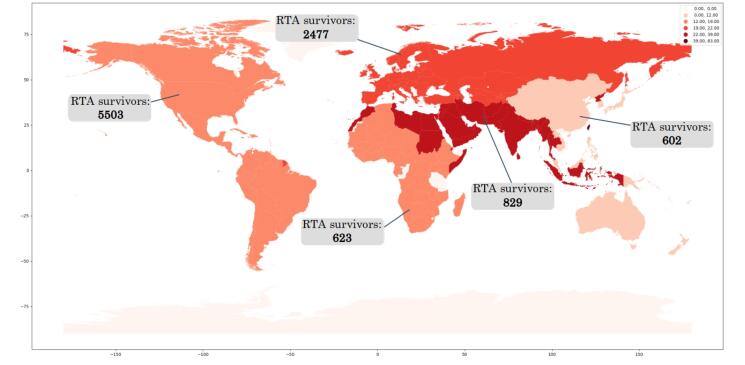
The distribution of RTA survivors on a global scale

 According to the subgroup analysis based on the different countries within the clinician-administered group, the most published papers were from the USA (n = 14 studies, with a prevalence of 23.8% (95% CI: 16.2%-33.6.2%; I^2^: 98.8%). According to our findings, the lowest prevalence was observed in Switzerland (5.8%), Germany (6%), and Japan (8%) in one, one, and three eligible studies. In contrast, Spain (39%), China (32.2%), Iran (30.5%), Greece (29.3%), and France (28.9%) had the highest prevalence in two, three, one, two, and four eligible studies, respectively (Figure 5). Furthermore, the distribution of RTA survivors is schematically presented in Figure 6.

 In subgroup analysis to show the prevalence of PTSD based on the injury severity within the clinician-administered group, in hospitalized patients, the prevalence rate was 18.7% (95% CI: 12.7%-26.6%); in cases attended to the emergency department, this rate was 16.7% (95% CI: 10.7%-25.2%) and in serious injuries 20.3% (95% CI: 14%-28.7%) (Supplementary file 2, Figure S1).

 In subgroup analysis based on age within the clinician-administered group, the prevalence of PTSD in adults (age > 18 years) was 19.2% (95% CI: 16.4%-22.4%; I^2^: 90.10%), and in children (age < 18 years) was 17.4% (95% CI: 11.9%-24.8%; I^2^: 96.10) (Figure S2). After removing outlier studies, the prevalence was estimated to be 17.8% (95%CI: 15.2%-20.8%; I^2^: 88.88%) in adults.

Figure S3 represents the result of the subgroup analysis based on the checklist used for diagnosis.

####  Self-Reported Measures 

 For self-reported PTSD, the prevalence rate was 22.8% (95% CI: 18.8%-27.3%; I^2^: 93.92%); After removing outliers, the total prevalence was decreased to 20.8% (95% CI: 17.5%-24.4%; I^2^: 91.51 %) using self-reported questionnaires (Figure 2 and Figure S4).

 In subgroup analysis based on the time points in self-reported group studies, 1 month after RTA, the prevalence of PTSD was 22.6% (95% CI: 15.9%-31.2%; I^2^: 92.61%) among 10 eligible studies; in 3 months following RTA, this rate was 19.7% (95% CI: 15.2%-25%; I^2^: 58.08) in 5 included studies, and at six months was 17.4% (95% CI: 15.8%-23%; I^2^: 83.06%) in the 10 included studies. At the other time point, consisting of 2 months following RTA, this rate was 53.4% (95% CI: 36.5%-69.6%; I^2^: 97.27%) in 3 eligible studies, and at 4 months was 19.6% (95% CI: 15.8%-33.6%%; I^2^: 54.69%) in two eligible studies (Figure S5).

 In subgroup analysis based on the WHO regions in self-reported group studies, the prevalence in American areas was 31.6% (95% CI: 29.9%-33.4%) among five studies; in European regions, 24.4% (95% CI: 22.7%-26.1%) in 18 eligible studies, 5.4% (95% CI: 1.7%-15.4%) among two related studies in Western Pacific regions, 25.5% in Asia (95% CI: 21.8%-29.7%) in 5 eligible studies, and 27.5% (95% CI: 24.9%-30.3%) in 3 African studies (Figure S6).

 According to the subgroup analysis based on the different countries, the most published papers were from the UK (n = 5 studies, with a prevalence of 22.1% (95% CI: 18.1%-26.2%; I^2^: 23.19%). According to our findings, the lowest prevalence was observed in Australia (5.4%), Malaysia (7.4%), and Portugal (10.2%) in eligible studies in each country. In contrast, Taiwan (82 %), Greece (78%), and the USA (32.7%) had the highest prevalence in one, one, and four eligible studies, respectively (Figure S7).

 We performed a subgroup analysis to show the prevalence of PTSD based on the injury severity. The results showed that in hospitalized patients, prevalence rates of PTSD following an RTA varied considerably across studies, ranging from 9% to 43.1% (point estimate of 21.5%); in cases attended to the emergency department, this rate was 19.7% (95% CI: 14.8%-25.8%). The prevalence rates ranged from 14.8% to 36.8% (point estimate of 24%) in the studies that included severe injury cases (Figure S8).

 In subgroup analysis based on age within the self-reported group, the prevalence of PTSD in adults (age > 18 years) was 23.3% (95% CI: 18.8%-28.5%), and in children (age < 18 years) was 21.5% (95% CI: 16.4%-27.6%) (Figure S9).

####  Combining the Studies with Clinician-Administered Measures and a Self-Reported Checklist for PTSD Assessment

 To evaluate the publication bias, a funnel plot was drawn, and according to the results of the Egger’s regression test, there was significant publication bias among the included studies (*P* < 0.001) (Figure S10).

 Meta-regression models were used to investigate further the contribution of a variable to the prevalence heterogeneity. The results of this multivariate analysis suggested that the mean age of RTA survivors was not associated with significant heterogeneity between prevalence rates of PTSD (*P* = 0.711) (Table 2, Figure S11).

**Table 2:** Multivariate meta-regression of included studies

**Meta-regression **			**Number of observations=24**		
REML estimate of between-study variance			tau2 = 0.01963		
% Residual variation due to heterogeneity			I-squared res: 94.34%		
Proportion of between-study variance explained			Adj R-squared = -1.62%		
**ES**	**exp(b) **	**SE **	**t **	***P*** **>|t|**	**[95% Confidence Interval]**
Mean age	-0.0008	0.0022	-0.37	0.711	[-0.0052, 0.0035]
cons	0.2276	0.0755	3.01	0.004	[0.0764, 0.3787]

###  Methodological Quality

 Eligible studies were reviewed using the JBI Evidence Quality Evaluation Checklist. The results show that all included studies were of moderate to high quality (more than 60% “yes” response). The detailed results of the quality assessment are presented in Table 3.

**Table 3:** Methodological quality assessment using the JBI Evidence Quality Evaluation Checklist (https://jbi.global/critical-appraisal-tools)

	**Q1**	**Q2**	**Q3**	**Q4**	**Q5**	**Q6**	**Q7**	**Q8**	**Q9**	**Q10**	**Q11**	**Q12**	**Q13**
**Cohort studies **													
Angerpointner (2020)^26^	NA	NA	Yes	Unclear	No	Unclear	Yes	Yes	Yes	Yes	Unclear		
Fitzharris (2006)^42^	NA	NA	Yes	Unclear	No	Unclear	Yes	Yes	Yes	Yes	Unclear		
Bryant (2003)^32^	Yes	Yes	Yes	Yes	Yes	Yes	Yes	Yes	Yes	Yes	Yes		
Ehring (2008)^40^	Yes	Yes	Yes	Yes	Yes	Yes	Yes	Yes	Yes	Yes	Yes		
Chossegros (2011)^35^	Yes	Yes	Yes	Yes	Yes	Yes	Yes	Yes	Yes	Yes	Yes		
Li (2021)^62^	Yes	Yes	Yes	Yes	Yes	Yes	Yes	Yes	Yes	Yes	Yes		
Coronas (2011)^36^	Yes	Yes	Yes	Yes	Yes	Yes	Yes	Yes	Yes	Yes	Yes		
Delahanty (2003)^37^	Yes	Yes	Yes	Yes	Yes	Yes	Yes	Yes					
Doohan (2017)^38^ (bus)	NA	NA	NA	NA	NA	NA	NA	NA	NA	NA	NA		
Ehlers (1998)^39^	NA	NA	Yes	Unclear	Unclear	Unclear	Yes	Yes	Yes	Yes	Yes		
Flesher (2001)^43^	Yes	Yes	Yes	Yes	Yes	Yes	Yes	Yes					
Fekadu (2019)^41^	Yes	Yes	Yes	Yes	Yes	Yes	Yes	Yes	Yes	Yes	Yes		
Kessler (2021)^108^	Yes	Yes	Yes	Yes	Yes	Yes	Yes	Yes	Yes	Yes	Yes		
Fredman (2017)^44^	Yes	Yes	Yes	Yes	Yes	Yes	Yes	Yes	Yes	Yes	Yes		
Frommberger (1998)^45^	Yes	Yes	Yes	Yes	Yes	Yes	Yes	Yes	Yes	Yes	Yes		
Fuglsang (2004)^46^	Yes	Yes	Yes	Yes	Yes	Yes	Yes	Yes	Yes	Yes	Yes		
Fullerton (2001)^47^	Yes	Yes	Yes	Yes	Yes	Yes	Yes	Yes	Yes	Yes	Yes		
Giannopoulou (2021)^106^	Yes	Yes	Yes	Yes	Yes	Yes	Yes	Yes	Yes	Yes	Yes		
Ziobrowski (2021)^107^	Yes	Yes	Yes	Yes	Yes	Yes	Yes	Yes	Yes	Yes	Yes		
Hamanaka (2006)^4^	Yes	Yes	Yes	Yes	Yes	Yes	Yes	Yes	Yes	Yes	Yes		
Harvey (1998)^49^	Yes	Yes	Yes	Yes	Yes	Yes	Yes	Yes	Yes	Yes	Yes		
Holeva (2001)^50^	Yes	Yes	Yes	Yes	Yes	Yes	Yes	Yes	Yes	Yes	Yes		
Hu (2018)^51^	Yes	Yes	Yes	Yes	Yes	Yes	Yes	Yes	Yes	Yes	Yes		
Kovacevic (2021)^59^	Yes	Yes	Yes	Yes	Yes	Yes	Yes	Yes	Yes	Yes	Yes		
Jeavons (2000)^54^	NA	NA	Yes	Yes	Yes	Yes	Yes	Yes	Yes	Yes	Yes		
Jones (2005)^55^	Yes	Yes	Yes	Yes	Yes	Yes	Yes	Yes	Yes	Yes	Yes		
Yrondi (2022)^111^	Yes	Yes	Yes	Yes	Yes	Yes	Yes	Yes	Yes	Yes	Yes		
Kassam-Adams (2009)^56^	Yes	Yes	Yes	Yes	Yes	Yes	Yes	Yes	Yes	Yes	Yes		
Bahari (2017)^27^	Yes	Yes	Yes	Unclear	Unclear	Unclear	Yes	Yes	Yes	Yes	Unclear		
Irish (2011)^52^	Yes	Yes	Yes	Yes	Yes	Yes	Yes	Yes	Yes	Yes	Yes		
Gabert-Quillen (2012)^48^	Yes	Yes	Yes	Yes	Yes	Yes	Yes	Yes	Yes	Yes	Yes		
Kobayashi (2019)^58^	NA	NA	Yes	Yes	Yes	Yes	Yes	Yes	Yes	Yes	Yes		
Kuhn (2006)^60^	Yes	Yes	Yes	Unclear	Unclear	Unclear	Yes	Yes	Yes	Yes	Unclear		
Schäfer (2006)^7^	Yes	Yes	Yes	Yes	Yes	Yes	Yes	Yes	Yes	Yes	Yes		
Yasan (2009)^83^	NA	NA	Yes	Yes	Yes	Yes	Yes	Yes	Yes	Yes	Yes		
Allenou (2010)^95^	Yes	Yes	Yes	Yes	Yes	Yes	Yes	Yes	Yes	Yes	Yes		
Mayou (1993)^64^	NA	NA	Yes	Unclear	Unclear	Unclear	Yes	Yes	Yes	Yes	Unclear		
Bryant (2000)^33^	Yes	Yes	Yes	Unclear	Unclear	Unclear	Yes	Yes	Yes	Yes	Unclear		
Bryant (2004)^90^	NA	NA	Yes	Unclear	Unclear	Unclear	Yes	Yes	Yes	Yes	Unclear		
Buckley (2004)^34^	NA	NA	Yes	Unclear	Unclear	Unclear	Yes	Yes	Yes	Yes	Unclear		
DI Gallo (1997)^91^	NA	NA	Yes	Unclear	Unclear	Unclear	Yes	Yes	Yes	Yes	Unclear		
Gillies (2003)^92^	NA	NA	Yes	Unclear	Unclear	Unclear	Yes	Yes	Yes	Yes	Unclear		
Brand (2014)^31^	NA	NA	Yes	Unclear	Unclear	Unclear	Unclear	Yes	Unclear	Unclear	Unclear		
Kim (2013)^87^ (subway drivers)	NA	NA	Yes	Yes	Yes	Yes	Yes	Yes	Yes	Yes	Yes		
Kovacevic (2021)^59^	Yes	Yes	Yes	Yes	Yes	Yes	Yes	Yes	Yes	Yes	Yes		
Landolt (2005)^94^ (PTSS)	Yes	Yes	Yes	Yes	Yes	Yes	Yes	Yes	Yes	Yes	Yes		
Maeda (2009)^105^ (ship)	Yes	Yes	Yes	Unclear	Unclear	Unclear	Yes	Yes	Yes	Yes	Unclear		
Matsuoka (2008)^63^	NA	NA	Yes	Yes	Yes	Yes	Yes	Yes	Yes	Yes	Yes		
Mayou (1997)^65^	Yes	Yes	Yes	Yes	Yes	Yes	Yes	Yes	Yes	Yes	Yes		
McFarlane (1997)^66^	Yes	Yes	Yes	Unclear	Unclear	Unclear	Yes	Yes	Yes	Yes	Unclear		
Suliman (2014)^78^	NA	NA	Yes	Yes	Yes	Yes	Yes	Yes	Yes	Yes	Yes		
Mehnert (2012)^89^	NA	NA	Yes	Yes	Yes	Yes	Yes	Yes	Yes	Yes	Yes		
Meiser-Stedman (2009)^96^	Yes	Yes	Yes	Yes	Yes	Yes	Yes	Yes	Yes	Yes	Yes		
Papadakaki (2017)^72^	Yes	Yes	Yes	Yes	Yes	Yes	Yes	Yes	Yes	Yes	Yes		
Naim (2014)^67^	Yes	Yes	Yes	Yes	Yes	Yes	Yes	Yes	Yes	Yes	Yes		
Nightingale (2000)^68^	NA	NA	Yes	Yes	Yes	Yes	Yes	Yes	Yes	Yes	Yes		
Nishi (2013)^70^	NA	NA	Yes	Yes	Yes	Yes	Yes	Yes	Yes	Yes	Yes		
Mirza (1998)^97^	Yes	Yes	Yes	Unclear	Yes	Yes	Yes	Yes	Yes	Yes	Yes		
Pervanidou (2007)^98^	Yes	Yes	Yes	Yes	Yes	Yes	Yes	Yes	Yes	Yes	Yes		
Ryb (2009) ^75^	Yes	Yes	Yes	Yes	Yes	Yes	Yes	Yes	Yes	Yes	Yes		
Shaikh (2012)^77^	Yes	Yes	Yes	Yes	Yes	Yes	Yes	Yes	Yes	Yes	Yes		
Smith (2007)^3^	Yes	Yes	Yes	Unclear	Unclear	Unclear	Yes	Yes	Yes	Yes	Unclear		
Stallard (2001)^100^	Yes	Yes	Yes	Unclear	Unclear	Unclear	Yes	Yes	Yes	Yes	Unclear		
Stallard (2004)^101^	Yes	Yes	Yes	Unclear	Unclear	Unclear	Yes	Yes	Yes	Yes	Unclear		
Sun (2013) ^79^	Yes	Yes	Yes	Yes	Yes	Yes	Yes	Yes	Yes	Yes	Yes		
Lesaca (1996)^85^	Yes	Yes	Yes	Unclear	Unclear	Unclear	Yes	Yes	Yes	Yes	Unclear		
Vaiva (2003)^81^	Yes	Yes	Yes	Unclear	Unclear	Unclear	Yes	Yes	Yes	Yes	Unclear		
Wang (2005)^82^	Yes	Yes	Yes	Yes	Yes	Yes	Yes	Yes	Yes	Yes	Yes		
Wu (2016)^103^	Yes	Yes	Yes	Yes	Yes	Yes	Yes	Yes	Yes	Yes	Yes		
**Cross-sectional **													
Iteke (2011)^53^	Yes	Yes	Yes	Yes	Unclear	Unclear	Yes	Yes					
AlShardan (2020)^25^	Yes	Yes	Yes	Yes	Unclear	Unclear	Yes	Yes					
Bedaso (2020)^28^	Yes	Yes	Yes	Yes	Yes	Yes	Yes	Yes					
Berna (2012)^16^	Yes	Yes	Yes	Yes	Unclear	Unclear	Yes	Yes					
Arora (2021)^110^	Yes	Yes	Yes	Yes	Yes	Yes	Yes	Yes					
Khodadadi-Hassankiadeh (2017)^57^	Yes	Yes	Yes	Yes	Unclear	Unclear	Yes	Yes					
Blanchard (1995)^30^	Yes	Yes	Yes	Yes	Yes	Yes	Yes	Yes					
Bezabh (2018)^29^	Yes	Yes	Yes	Yes	Yes	Yes	Yes	Yes					
Kupchik (2007)^61^	Yes	Yes	Yes	Yes	Yes	Yes	Yes	Yes					
Ongecha-Owuor (2004)^71^	Yes	Yes	Yes	Yes	Yes	Yes	Yes	Yes					
Neylan (2021)^112^	Yes	Yes	Yes	Yes	Yes	Yes	Yes	Yes					
Ning (2017)^69^	Yes	Yes	Yes	Yes	Yes	Yes	Yes	Yes					
Yohannes (2018)^84^	Yes	Yes	Yes	Yes	Yes	Yes	Yes	Yes					
Williams (2015)^102^	Yes	Yes	Yes	Yes	Yes	Yes	Yes	Yes					
Saberi (2013) ^76^	Yes	Yes	Yes	Yes	Unclear	Unclear	Yes	Yes					
Pires (2013)^74^	Yes	Yes	Yes	Yes	Unclear	Unclear	Yes	Yes					
Prakasam (2013)^73^	Yes	Yes	Yes	Yes	Yes	Yes	Yes	Yes					
Salter (2004)^99^	Unclear	Unclear	Unclear	Unclear	Unclear	Unclear	Unclear	Unclear					
**Case-control**													
Jones-Alexander (2005)^93^	Yes	Yes	Yes	Yes	Yes	Yes	Yes	Yes	Yes	Unclear			
Ursano (1999)^80^	Unclear	No	Yes	Yes	Yes	Yes	Yes	Yes	Yes	Yes			
**RCT **													
Zehnder (2010)^104^	NA	NA	NA	NA	NA	NA	NA	NA	NA	NA	NA	NA	NA
**Train accident **													
Lemos (2018)^88^	Yes	Yes	Yes	Yes	Yes	Yes	Yes	Yes	Yes	Yes	Yes		
Engelhard (2002)^86^	Yes	Yes	Yes	Unclear	Unclear	Yes	Yes	Yes	Yes	Unclear	Yes		

## Discussion

 Our comprehensive meta-analysis, encompassing 82 studies identified from an initial 11,142 articles, revealed an overall pooled prevalence of PTSD of 20.3% (95% CI: 18.1%-22.8%) among the studied population. We observed a slight variation based on assessment methods, with clinician-administered assessments indicating an 18.7% prevalence (95% CI: 16.0%-21.8%) and self-reported questionnaires showing 22.8% (95% CI: 18.8%-27.3%). After removing outliers, these rates were adjusted to 18.1% (95% CI: 15.4%-21.0%) for clinician-administered assessments and 20.8% (95% CI: 17.5%-24.4%) for self-reported questionnaires. A significant temporal pattern was identified, with PTSD prevalence peaking at 29.4% (95% CI: 22.4%-37.5%) one-month post-RTA, subsequently decreasing to 18.8% (95% CI: 14.8%-23.5%) at three months (*P* < 0.001). Interestingly, age was not a significant predictor of PTSD prevalence rates. We also noted considerable geographic variability in PTSD prevalence, with lower rates observed in Switzerland, Australia, Germany, and Japan, compared to higher rates in Spain, China, and Iran. The included studies were assessed to be of moderate to high quality according to Joanna Briggs Institute standards, ensuring the reliability of these findings. The results of this systematic review and meta-analysis provide a comprehensive overview of the prevalence of PTSD among survivors of RTAs across various geographical regions and assessment methods. The study included data from multiple countries and employed a range of diagnostic tools, resulting in a PTSD prevalence rate ranging from 2.9% to 77.8%. Furthermore, the subgroup analysis revealed that the assessment tools most frequently utilized were PCL-C, CAPS, and SCID. The wide prevalence rate of PTSD might be related to variances in the time interval between the trauma’s occurrence and the assessment of PTSD. Factors such as the parameters used to diagnose this disorder and sample characteristics, including gender, type of RTA, and severity of injury, may also influence the PTSD prevalence rates.

 Additionally, variations in social support, family stability, and parental involvement may contribute to differences in PTSD prevalence among participants. In a previously published systematic review in 2013, the incidence of PTSD varied from 6 to 45% depending on the type of accident, community support, the severity of the stress, and a history of mental illness.^19^ In a recent systematic review of the delayed PTSD prevalence with the current study team, the total prevalence was 13.5%,^113^ and in term of risk factors associated with PTSD, Sabahi et al noted that several factors, including female gender, pre-traumatic depression, a history of RTA, peritraumatic dissociative experiences, a diagnosis of ASD, rumination, greater injury severity, and engagement in litigation or compensation following the traumatic event, were significant predictors of PTSD.^18^

 It is essential to obtain a reliable estimation of PTSD prevalence without adjusting for age following an RTA. This approach will help mental health professionals accurately identify the number of adolescents and children at risk for the disorder and effectively allocate resources for prevention and treatment interventions. A meta-analysis with a total of 1532 children and adolescents reported that one-fifth of children and adolescents who participated in RTAs later developed PTSD, underscoring the importance of regular PTSD assessments and the implementation of timely psychological interventions for this vulnerable group.^114^

 The adverse psychological repercussions resulting from MVA are significant, with all studies indicating detrimental effects on at least one aspect of psychopathology.^115^ Failure to address these symptoms may result in a significant risk of progression to severe mental health disorders, including major depressive disorder (MDD), PTSD, panic disorder, and generalized anxiety disorder.^116,117^ A systematic review conducted by Marasini and colleagues revealed a significant prevalence of adverse psychological outcomes after an MVA. The findings demonstrated a consistent pattern, highlighting the predominance of specific psychopathologies, including PTSD, depression, anxiety, travel-related phobia, and emotional distress.^115^ While Injuries significantly contribute to detrimental mental health outcomes, Individuals who have not entirely recovered from their accidents, along with those who have suffered severe injuries, display a markedly higher likelihood of experiencing adverse psychological effects.^17,118^

 It is imperative to underscore the significance of two prior studies regarding the prevalence and predictors of PTSD among survivors of RTA. The first study conducted by Heron Delaney et al^19^ explored various potential predictors of subsequent PTSD following RTA. The authors identified several contributing factors, including rumination related to the traumatic experience, a perceived sense of imminent danger, insufficient social support, the heightened intensity of ASD symptoms, ongoing physical ailments, historical and emotional difficulties, previous anxiety disorders, and participation in legal proceedings or compensation processes, all of which serve as reliable indicators of PTSD.^19^ Additionally, a second study conducted by Lin et al presented a meta-analysis encompassing 15 highly heterogeneous studies involving 6,804 RTA survivors, whereby a pooled prevalence rate of 22.25% (95% confidence interval: 16.71%–28.33%) was estimated.^15^ Moreover, subgroup analyses indicated that the prevalence of PTSD among RTA survivors exhibited considerable variation across studies, influenced by factors such as the PTSD assessment tool utilized, geographic location, ethnicity, gender, and educational level.

###  Variability in PTSD Prevalence

####  Clinician-Administered Measures

 The range of PTSD prevalence using these measures is striking, from single-digit percentages to over 80% at some points. While some variation is expected due to differing study populations and methodologies, such extreme differences suggest that other factors are at play. For example, the Lesaca et al^85^ study reported 48% in one month, and the Kobayashi et al^58^ study reported 81% in six weeks are exceptionally high. These outlier results may be due to unique characteristics of the study samples (e.g., severity of injuries, pre-existing mental health conditions, cultural context) or specific study methodologies. Conversely, studies like Ongecha-Owuor et al,^71^ with 13.3% at one month and the study reporting 1.7% at six months, represent the lower end of the spectrum, possibly reflecting more resilient populations or differences in how PTSD was diagnosed.

####  Self-Report Questionnaires

 Similar to clinician-administered measures, self-report data also demonstrates substantial variability. The Allenou et al^95^ study’s low prevalence of 3.6% contrasts sharply with the Fekadu et al^41^ study’s 46.5%. Again, differences in study populations, cultural factors, the specific self-report tool used, and the timing of assessment likely contribute to this variation. The Wang et al^82^ study’s extremely high rate of 82.8% at six weeks is a clear outlier that requires further investigation. While the authors suggest a potential link between riding motor scooters and experiencing greater emotional stress (citing the higher prevalence of scooter riders in their sample and Murray et al’s^119^ findings), this alone may not fully account for such a dramatic difference. The Wang et al^82^ study also found a high rate of ASD at one week (72%), which, while similar to Murray et al, was significantly higher than Jaspers’ results.^120^ The authors acknowledge that while ASD may be present early on, it doesn’t always develop into PTSD. They emphasize the importance of considering various contributing factors, including biological predispositions, pre-existing psychosocial factors, post-accident events, and, crucially, the individual’s subjective experience of the trauma. They rightly point out that the subjective meaning of the event could be a powerful predictor of PTSD development. Therefore, while the high prevalence of scooter riders in the Wang et al study^82^ might contribute to their findings, it’s likely a complex interplay of factors, including the subjective impact of the accident, that underlies their reported 82.8% PTSD rate.

 Our results showed that in the time to measure PTSD between 1 to 6 months after RTAs, the overall prevalence varies across studies, ranging from 8% to 36%. This rate decreased when we performed a subgroup analysis on the studies that evaluated PTSD between 4 and 6 months after RTA, varying from 18% to 28%.%. However, we should consider that only limited studies were eligible to be included in the latter subgroup analysis compared to those in the first subgroup. In addition, to determine the prevalence of PTSD, all related studies on whether cases were admitted to the emergency department and outpatients were included in the meta-analysis. The analysis results showed that a higher prevalence of PTSD was observed in cases with serious injury (20.3%). In comparison, 31% of hospitalized patients following RTA met PTSD criteria at the time point of 1-6 months. The rate was 20% for those attending the emergency department cases.

 The present study systematically assessed the prevalence among adult and adolescent survivors of RTAs. The results indicated older RTA victims (21.5%) were more susceptible to PTSD than younger ones (17.4%). Evidence suggests that age-related stressors can intensify PTSD symptoms. Factors such as role and functional changes accompanying aging—retirement, bereavement, declining physical health that limits autonomy, and reduced social support may contribute to an increase in PTSD symptoms.^121^ A comprehensive subgroup analysis was conducted, considering various temporal dimensions across distinct age categories and the severity of injuries—differentiating between severe and life-threatening injuries versus minor injuries and outpatient conditions.

 Here, we noticed the difference between self-reported (20.3%) and clinician-based PTSD prevalence (22.8%) results that stem from various factors. Self-reports can be influenced by subjective biases, emotional states, or limited understanding of PTSD symptoms, leading to over- or underestimation. Clinicians, by contrast, use structured interviews and standardized diagnostic criteria, providing greater precision. Survivors may interpret symptoms differently in self-reports or struggle to distinguish PTSD from other conditions. Additionally, rapport with clinicians can affect disclosure during interviews, while cultural or language barriers may influence the accuracy of both methods. Severe symptoms can also impair insight, affecting self-reports, whereas clinicians can contextualize symptoms more effectively. Combining both approaches enhances diagnostic accuracy and understanding.^122-124^

 Additionally, the analysis was stratified by World Health Organization (WHO) regions and specific countries to enhance the contextual understanding of PTSD prevalence. However, this study faced some limitations. Notably, it did not incorporate research with delayed PTSD assessments. This might have implications for the comprehensiveness of our findings. Furthermore, the lack of a gender-specific subgroup analysis limits the capacity to make detailed conclusions about how gender affects PTSD outcomes. Furthermore, because few studies report PTSD rates among survivors based on their position in the vehicle, analyzing this variable was not possible.

## Recommendations for Future Studies

 Based on the findings of this study, several clinical recommendations can be made to enhance the identification and management of PTSD among RTA survivors. Firstly, it is crucial to implement routine screening for PTSD among RTA survivors. The early utilization of validated assessment tools, particularly during the initial months following the accident, facilitates the timely identification of high-risk individuals. However, the precise implication of therapeutic interventions in self-reported PTSD victims is necessary to avoid overdiagnosis and overtreatment. Furthermore, practical psychological support services should be integrated into treatment plans, encompassing Cognitive Behavioral Therapy and family counseling. Given the variability of PTSD prevalence rates and demographic factors, interventions must be tailored accordingly. Healthcare providers need to be adequately developed to enhance their understanding of PTSD and its potential manifestations in survivors, particularly at the primary level of care.

 To enhance the understanding of PTSD among RTA survivors, future studies should concentrate on various demographic groups. Given the limited research on PTSD in children and adolescents, they should prioritize these populations. Then, more epidemiological research can clarify the explicit causal relationships between factors, leading to targeted preventive strategies. By examining the relationship between PTSD and other psychological disorders like depression and anxiety, valuable insights will be gained for developing treatment strategies that encompass various aspects of mental health in survivors of RTAs. Studies should evaluate the effectiveness and differences between self-reported and clinically administered approaches to suggest a reliable and optimized strategy. Finally, the enhanced understanding and management of survivors lead to improved recovery and quality of life.

## Conclusion

 The prevalence of PTSD among survivors was measured at 20.3%. Countries such as Switzerland, Australia, Germany, and Japan exhibited the lowest prevalence rates, whereas Spain, China, and Iran recorded the highest prevalence rates. Moreover, the subgroup analysis indicated that the frequently employed checklists and criteria for the assessment of PTSD included the PCL-C, the CAPS, and the SCID. The severity of injuries sustained significantly influenced the prevalence of PTSD. Future epidemiological studies are warranted to investigate potential causal relationships between the positioning of individuals within vehicles and the development of PTSD. This exploration aims to enhance our comprehension of the determinants of PTSD and subsequently improve prevention and treatment strategies for survivors of RTAs.

## Competing Interests

 None.

## Data Availability Statement

 Not applicable.

## Ethical Approval

 The regional ethics committee of Tabriz University of Medical Sciences approved this study (IR.TBZMED.REC.1400.966).

## Supplementary Files


Supplementary file 1. Search strategy of PubMed (PTSD + Traffic Accident).

Supplementary file 2 contains Figures S1-S11.
