# Supplementary material for: A systematic review and meta-analysis of the prevalence of post-traumatic stress disorder (PTSD) in road traffic accident survivors
Source: Health Promot Perspect. 2025 Nov 4;15(3):213–35. doi: 10.34172/hpp.025.43651 (PMC12680517; doi:10.34172/hpp.025.43651)
Supplement: Supplementary file 1 — Search strategy of PubMed (PTSD + Traffic Accident). [file hpp-15-213-s001.pdf]

Shahsavarinia et al, **Health Promotion Perspectives.** 2025;15(3):213-S1
doi: 10.34172/hpp.025.43651
https://hpp.tbzmed.ac.ir

Supplementary file 1. Search Strategy of PubMed (PTSD + Traffic Accident)

Search	Query
#1	Search: "Stress Disorders, Post-Traumatic"
#2	Search: (((((((PTSD[Title/Abstract]) OR ("Moral Injur*"[Title/Abstract])) OR ("Posttraumatic Neuroses"[Title/Abstract])) OR ("Post traumatic Neuroses"[Title/Abstract])) OR ("Post-traumatic Neuroses"[Title/Abstract])) OR ("Posttraumatic Stress Disorder*"[Title/Abstract])) OR ("Post traumatic Stress Disorder*"[Title/Abstract])) OR ("Post-traumatic Stress Disorder*"[Title/Abstract])
#3	Search: (("Stress Disorders, Post-Traumatic") OR (((((((PTSD[Title/Abstract]) OR ("Moral Injur*"[Title/Abstract])) OR ("Posttraumatic Neuroses"[Title/Abstract])) OR ("Post traumatic Neuroses"[Title/Abstract])) OR ("Post-traumatic Neuroses"[Title/Abstract])) OR ("Posttraumatic Stress Disorder*"[Title/Abstract])) OR ("Post traumatic Stress Disorder*"[Title/Abstract]))) OR ("Post-traumatic Stress Disorder*"[Title/Abstract])
#4	Search: "Accidents, Traffic"
#5	Search: (((((((((("Traffic Accident*"[Title/Abstract]) OR ("Traffic Collision*"[Title/Abstract])) OR ("Traffic Crash*"[Title/Abstract])) OR ("road accident*"[Title/Abstract])) OR ("motorcycle accident*"[Title/Abstract])) OR ("motorcar accident*"[Title/Abstract])) OR ("motor vehicle accident*"[Title/Abstract])) OR (accident*[Title/Abstract])) OR (incident[Title/Abstract])) OR ("road traffic"[Title/Abstract])) OR ("motorcycle traffic accident*"[Title/Abstract])
#6	Search: ("Accidents, Traffic") OR ((((((((((("Traffic Accident*"[Title/Abstract]) OR ("Traffic Collision*"[Title/Abstract])) OR ("Traffic Crash*"[Title/Abstract])) OR ("road accident*"[Title/Abstract])) OR ("motorcycle accident*"[Title/Abstract])) OR ("motorcar accident*"[Title/Abstract])) OR ("motor vehicle accident*"[Title/Abstract])) OR (accident*[Title/Abstract])) OR (incident[Title/Abstract])) OR ("road traffic"[Title/Abstract])) OR ("motorcycle traffic accident*"[Title/Abstract]))
#7	Search: (("Accidents, Traffic") OR ((((((((((("Traffic Accident*"[Title/Abstract]) OR ("Traffic Collision*"[Title/Abstract])) OR ("Traffic Crash*"[Title/Abstract])) OR ("road accident*"[Title/Abstract])) OR ("motorcycle accident*"[Title/Abstract])) OR ("motorcar accident*"[Title/Abstract])) OR ("motor vehicle accident*"[Title/Abstract])) OR (accident*[Title/Abstract])) OR (incident[Title/Abstract])) OR ("road traffic"[Title/Abstract])) OR ("motorcycle traffic accident*"[Title/Abstract]))) AND ((("Stress Disorders, Post-Traumatic") OR (((((((PTSD[Title/Abstract]) OR ("Moral Injur*"[Title/Abstract])) OR ("Posttraumatic Neuroses"[Title/Abstract])) OR ("Post traumatic Neuroses"[Title/Abstract])) OR ("Post-traumatic

Search	Query
	Neuroses"[Title/Abstract])) OR ("Posttraumatic Stress Disorder*"[Title/Abstract])) OR ("Post traumatic Stress Disorder*"[Title/Abstract]))) OR ("Post-traumatic Stress Disorder*"[Title/Abstract]))
