# Supplementary material for: A systematic review and meta-analysis of the prevalence of post-traumatic stress disorder (PTSD) in road traffic accident survivors
Source: Health Promot Perspect. 2025 Nov 4;15(3):213–35. doi: 10.34172/hpp.025.43651 (PMC12680517; doi:10.34172/hpp.025.43651)
Supplement: Supplementary file 2 — contains Figures S1-S11. [file hpp-15-213-s002.pdf]

Shahsavarinia et al, **Health Promotion Perspectives.** 2025;15(3):213-S2
doi: 10.34172/hpp.025.43651
https://hpp.tbzmed.ac.ir

Supplementary file 2

	Study name	Subgroup within study	Event rate	Lower limit	Upper limit	Z-Value	p-Value
Admitted to hospital	Blanchard (1995)	Admitted to hospital	0.392	0.319	0.470	-2.693	0.007
Admitted to hospital	Jeavons (2000)	Admitted to hospital	0.083	0.038	0.173	-5.624	0.000
Admitted to hospital	Jeavons (2000).	Admitted to hospital	0.080	0.034	0.179	-5.217	0.000
Admitted to hospital	Khodadadi-Hassankiadeh (2017)	Admitted to hospital	0.305	0.267	0.346	-8.717	0.000
Admitted to hospital	Salter (2004)	Admitted to hospital	0.370	0.263	0.491	-2.103	0.035
Admitted to hospital	Wu (2016)	Admitted to hospital	0.248	0.213	0.286	-11.113	0.000
Admitted to hospital			0.250	0.184	0.330	-5.496	0.000
Admitted to ICU	Nishi (2013)	Admitted to ICU	0.075	0.038	0.143	-6.813	0.000
Admitted to ICU			0.075	0.038	0.143	-6.813	0.000
Admitted to level-1 trauma centers	Kobayashi (2019)	Admitted to level-1 trauma centers	0.192	0.131	0.272	-6.200	0.000
Admitted to level-1 trauma centers	Kobayashi (2019).	Admitted to level-1 trauma centers	0.081	0.048	0.135	-8.382	0.000
Admitted to level-1 trauma centers	Kobayashi (2019)..	Admitted to level-1 trauma centers	0.141	0.085	0.224	-6.257	0.000
Admitted to level-1 trauma centers	Kobayashi (2019)...	Admitted to level-1 trauma centers	0.025	0.008	0.075	-6.213	0.000
Admitted to level-1 trauma centers			0.100	0.051	0.187	-5.939	0.000
Attended ED	Gillies (2003)	Attended ED	0.440	0.365	0.518	-1.505	0.132
Attended ED	Gillies (2003).	Attended ED	0.220	0.162	0.291	-6.590	0.000
Attended ED	Gillies (2003)..	Attended ED	0.070	0.039	0.122	-8.296	0.000
Attended ED	Mayou (1997)	Attended ED	0.100	0.056	0.171	-6.945	0.000
Attended ED	Mirza (1998)	Attended ED	0.120	0.073	0.192	-7.063	0.000
Attended ED	Mirza (1998) ..	Attended ED	0.034	0.013	0.087	-6.617	0.000
Attended ED	Mirza (1998).	Attended ED	0.017	0.004	0.065	-5.722	0.000
Attended ED	Stallard (2001)	Attended ED	0.371	0.281	0.471	-2.512	0.012
Attended ED	Stallard (2004)	Attended ED	0.291	0.226	0.367	-5.084	0.000
Attended ED	Yasan (2009)	Attended ED	0.298	0.210	0.404	-3.592	0.000
Attended ED	Yasan (2009).	Attended ED	0.231	0.151	0.337	-4.477	0.000
Attended ED			0.167	0.107	0.252	-6.071	0.000
Hospitalized	Berna (2012)	Hospitalized	0.077	0.044	0.131	-8.245	0.000
Hospitalized	Bryant (2000)	Hospitalized	0.210	0.145	0.295	-5.737	0.000
Hospitalized	Bryant (2003)	Hospitalized	0.220	0.145	0.319	-4.890	0.000
Hospitalized	Flesher (2001)	Hospitalized	0.170	0.099	0.276	-4.983	0.000
Hospitalized	Gabert-Quillen (2012)	Hospitalized	0.070	0.042	0.115	-9.357	0.000
Hospitalized	Harvey & Bryant (1998)	Hospitalized	0.254	0.166	0.367	-3.952	0.000
Hospitalized	Irish (2011)	Hospitalized	0.104	0.068	0.155	-9.206	0.000
Hospitalized	McFarlane (1997)	Hospitalized	0.269	0.134	0.467	-2.260	0.024
Hospitalized	Ryb (2009)	Hospitalized	0.275	0.232	0.323	-8.292	0.000
Hospitalized	Shaikh (2012)	Hospitalized	0.333	0.168	0.553	-1.500	0.134
Hospitalized	Shaikh (2012).	Hospitalized	0.389	0.198	0.622	-0.934	0.350
Hospitalized	Vaiva (2003)	Hospitalized	0.510	0.422	0.597	0.222	0.824
Hospitalized	Zehnder (2010)	Hospitalized	0.071	0.025	0.184	-4.670	0.000
Hospitalized	Zehnder (2010).	Hospitalized	0.040	0.010	0.146	-4.404	0.000
Hospitalized			0.187	0.127	0.266	-6.336	0.000
Mild	Hu (2018)	Mild	0.414	0.305	0.532	-1.432	0.152
Mild			0.414	0.305	0.532	-1.432	0.152
Mild, moderate & severe	Irish (2011).	Mild, moderate & severe	0.071	0.043	0.117	-9.248	0.000
Mild, moderate & severe			0.071	0.043	0.117	-9.248	0.000
Minor & Major	Mayou (1993)	Minor & Major	0.080	0.048	0.131	-8.740	0.000
Minor & Major	Suliman (2014)	Minor & Major	0.196	0.131	0.283	-5.714	0.000
Minor & Major	Suliman (2014).	Minor & Major	0.122	0.071	0.201	-6.492	0.000
Minor & Major			0.127	0.073	0.211	-6.160	0.000
Minor, admitted to ED	Naim (2014)	Minor, admitted to ED	0.068	0.047	0.096	-13.420	0.000
Minor, admitted to ED			0.068	0.047	0.096	-13.420	0.000
Moderate to severe	Ehring (2008)	Moderate to severe	0.121	0.077	0.186	-7.679	0.000
Moderate to severe	Kuhn (2006)	Moderate to severe	0.060	0.021	0.159	-4.977	0.000
Moderate to severe	Pervanidou (2007)	Moderate to severe	0.411	0.291	0.543	-1.325	0.185
Moderate to severe	Pervanidou (2007).	Moderate to severe	0.188	0.101	0.323	-3.960	0.000
Moderate to severe			0.170	0.072	0.351	-3.201	0.001
NM	Hannah N Ziobrowski (2021)	NM	0.266	0.240	0.294	-14.204	0.000
NM	Lesaca (1996)	NM	0.480	0.333	0.630	-0.256	0.798
NM	Lesaca (1996).	NM	0.240	0.134	0.393	-3.152	0.002
NM	Lesaca (1996)..	NM	0.140	0.044	0.365	-2.817	0.005
NM	Sun (2013)	NM	0.339	0.233	0.465	-2.489	0.013
NM			0.301	0.222	0.393	-4.031	0.000
None, mild & moderate	Ehlers (1998)	None, mild & moderate	0.231	0.204	0.260	-15.105	0.000
None, mild & moderate			0.231	0.204	0.260	-15.105	0.000
Outpatient clinic	Kupchik (2007)	Outpatient clinic	0.500	0.376	0.624	0.000	1.000
Outpatient clinic			0.500	0.376	0.624	0.000	1.000
Serious	Buckley (2004)	Serious	0.170	0.097	0.281	-4.802	0.000
Serious	Coronas (2011)	Serious	0.454	0.367	0.544	-1.002	0.316
Serious	Coronas (2011).	Serious	0.328	0.246	0.422	-3.499	0.000
Serious	Delahanty (2003)	Serious	0.200	0.117	0.321	-4.259	0.000
Serious	Engelhard (2002)	Serious	0.170	0.072	0.351	-3.207	0.001
Serious	Fullerton (2001)	Serious	0.344	0.265	0.432	-3.387	0.001
Serious	Hamanaka (2006)	Serious	0.085	0.041	0.168	-6.001	0.000
Serious	Jones (2005)	Serious	0.274	0.205	0.357	-4.974	0.000
Serious	Jones (2005).	Serious	0.304	0.231	0.388	-4.361	0.000
Serious	Jones (2005)..	Serious	0.180	0.123	0.255	-6.668	0.000
Serious	Jones (2005)...	Serious	0.172	0.117	0.246	-6.788	0.000
Serious	Matsuoka (2008)	Serious	0.080	0.041	0.152	-6.626	0.000
Serious	Ongecha-Owuor (2004)	Serious	0.133	0.097	0.180	-10.343	0.000
Serious	Ursano (1999)	Serious	0.344	0.265	0.432	-3.387	0.001
Serious	Ursano (1999).	Serious	0.252	0.176	0.347	-4.700	0.000
Serious	Ursano (1999)..	Serious	0.182	0.118	0.270	-5.770	0.000
Serious	Williams (2015)	Serious	0.074	0.066	0.083	-39.709	0.000
Serious			0.203	0.140	0.287	-5.898	0.000
Overall			0.207	0.190	0.224	-25.435	0.000

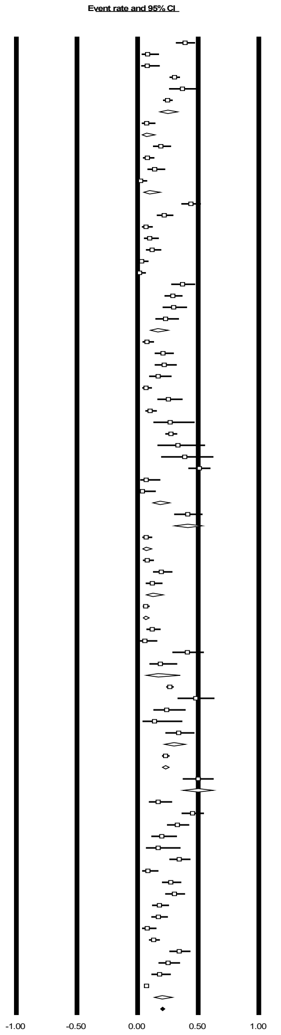

Figure S1. The forest plot for the prevalence of PTSD based on injury severity within Clinician-administered group.

Model	Group by Subgroup within study	Study name	Subgroup within study	Event rate	Lower limit	Upper limit	Z-Value	p-Value
	Adult	Berna (2012)	Adult	0.077	0.044	0.131	-8.245	0.000
	Adult	Blanchard (1995)	Adult	0.392	0.319	0.470	-2.693	0.007
	Adult	Bryant (2000)	Adult	0.210	0.145	0.295	-5.737	0.000
	Adult	Bryant (2003)	Adult	0.220	0.145	0.319	-4.890	0.000
	Adult	Buckley (2004)	Adult	0.170	0.097	0.281	-4.802	0.000
	Adult	Coronas (2011)	Adult	0.454	0.367	0.544	-1.002	0.316
	Adult	Coronas (2011).	Adult	0.328	0.246	0.422	-3.499	0.000
	Adult	Delahanty (2003)	Adult	0.200	0.117	0.321	-4.259	0.000
	Adult	Ehlers (1998)	Adult	0.231	0.204	0.260	-15.105	0.000
	Adult	Ehring (2008)	Adult	0.121	0.077	0.186	-7.679	0.000
	Adult	Engelhard (2002)	Adult	0.170	0.072	0.351	-3.207	0.001
	Adult	Flesher (2001)	Adult	0.170	0.099	0.276	-4.983	0.000
	Adult	Fullerton (2001)	Adult	0.344	0.265	0.432	-3.387	0.001
	Adult	Gabert-Quillen (2012)	Adult	0.700	0.633	0.759	5.505	0.000
	Adult	Hamanaka (2006)	Adult	0.085	0.041	0.168	-6.001	0.000
	Adult	Hannah N Ziobrowski (2021)	Adult	0.266	0.240	0.294	-14.204	0.000
	Adult	Harvey & Bryant (1998)	Adult	0.254	0.166	0.367	-3.952	0.000
	Adult	Hu (2018)	Adult	0.414	0.305	0.532	-1.432	0.152
	Adult	Irish (2011)	Adult	0.104	0.068	0.155	-9.206	0.000
	Adult	Irish (2011).	Adult	0.071	0.043	0.117	-9.248	0.000
	Adult	Jeavons (2000)	Adult	0.083	0.038	0.173	-5.624	0.000
	Adult	Jeavons (2000).	Adult	0.080	0.034	0.179	-5.217	0.000
	Adult	Khodadadi-Hassankiadeh (2017)	Adult	0.305	0.267	0.346	-8.717	0.000
	Adult	Kobayashi (2019)	Adult	0.192	0.131	0.272	-6.200	0.000
	Adult	Kobayashi (2019).	Adult	0.810	0.742	0.864	7.195	0.000
	Adult	Kobayashi (2019)..	Adult	0.141	0.085	0.224	-6.257	0.000
	Adult	Kobayashi (2019)...	Adult	0.025	0.008	0.075	-6.213	0.000
	Adult	Kuhn (2006)	Adult	0.060	0.021	0.159	-4.977	0.000
	Adult	Kupchik (2007)	Adult	0.500	0.376	0.624	0.000	1.000
	Adult	Lesaca (1996)	Adult	0.480	0.333	0.630	-0.256	0.798
	Adult	Lesaca (1996).	Adult	0.240	0.134	0.393	-3.152	0.002
	Adult	Lesaca (1996)..	Adult	0.140	0.044	0.365	-2.817	0.005
	Adult	Matsuoka (2008)	Adult	0.080	0.041	0.152	-6.626	0.000
	Adult	Mayou (1993)	Adult	0.080	0.048	0.131	-8.740	0.000
	Adult	Mayou (1997)	Adult	0.100	0.056	0.171	-6.945	0.000
	Adult	McFarlane (1997)	Adult	0.269	0.134	0.467	-2.260	0.024
	Adult	Naim (2014)	Adult	0.068	0.047	0.096	-13.420	0.000
	Adult	Nishi (2013)	Adult	0.075	0.038	0.143	-6.813	0.000
	Adult	Ongecha-Owuor (2004)	Adult	0.133	0.097	0.180	-10.343	0.000
	Adult	Ryb (2009)	Adult	0.275	0.232	0.323	-8.292	0.000
	Adult	Shaikh (2012)	Adult	0.333	0.168	0.553	-1.500	0.134
	Adult	Shaikh (2012).	Adult	0.389	0.198	0.622	-0.934	0.350
	Adult	Suliman (2014)	Adult	0.196	0.131	0.283	-5.714	0.000
	Adult	Suliman (2014).	Adult	0.122	0.071	0.201	-6.492	0.000
	Adult	Sun (2013)	Adult	0.339	0.233	0.465	-2.489	0.013
	Adult	Ursano (1999)	Adult	0.344	0.265	0.432	-3.387	0.001
	Adult	Ursano (1999).	Adult	0.252	0.176	0.347	-4.700	0.000
	Adult	Ursano (1999)..	Adult	0.182	0.118	0.270	-5.770	0.000
	Adult	Vaiva (2003)	Adult	0.510	0.422	0.597	0.222	0.824
	Adult	Yasan (2009)	Adult	0.298	0.210	0.404	-3.592	0.000
	Adult	Yasan (2009).	Adult	0.231	0.151	0.337	-4.477	0.000
Random	Adult			0.215	0.178	0.256	-10.956	0.000
	Children	Gillies (2003)	Children	0.440	0.365	0.518	-1.505	0.132
	Children	Gillies (2003).	Children	0.220	0.162	0.291	-6.590	0.000
	Children	Gillies (2003)..	Children	0.070	0.039	0.122	-8.296	0.000
	Children	Jones (2005)	Children	0.274	0.205	0.357	-4.974	0.000
	Children	Jones (2005).	Children	0.304	0.231	0.388	-4.361	0.000
	Children	Jones (2005)..	Children	0.180	0.123	0.255	-6.668	0.000
	Children	Jones (2005)...	Children	0.172	0.117	0.246	-6.788	0.000
	Children	Mirza (1998)	Children	0.120	0.073	0.192	-7.063	0.000
	Children	Mirza (1998) ..	Children	0.034	0.013	0.087	-6.617	0.000
	Children	Mirza (1998).	Children	0.017	0.004	0.065	-5.722	0.000
	Children	Pervanidou (2007)	Children	0.411	0.291	0.543	-1.325	0.185
	Children	Pervanidou (2007).	Children	0.188	0.101	0.323	-3.960	0.000
	Children	Salter (2004)	Children	0.370	0.263	0.491	-2.103	0.035
	Children	Stallard (2001)	Children	0.371	0.281	0.471	-2.512	0.012
	Children	Stallard (2004)	Children	0.291	0.226	0.367	-5.084	0.000
	Children	Williams (2015)	Children	0.074	0.066	0.083	-39.709	0.000
	Children	Wu (2016)	Children	0.248	0.213	0.286	-11.113	0.000
	Children	Zehnder (2010)	Children	0.071	0.025	0.184	-4.670	0.000
	Children	Zehnder (2010).	Children	0.040	0.010	0.146	-4.404	0.000
Random	Children			0.174	0.119	0.248	-6.852	0.000

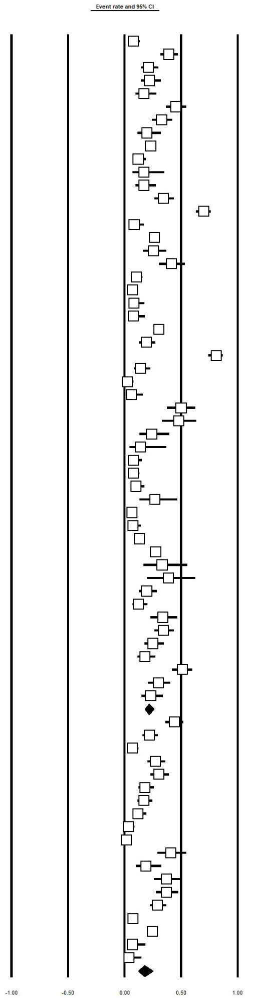

Figure S2. The forest plot for the prevalence of PTSD based on age within Clinician-administered group.

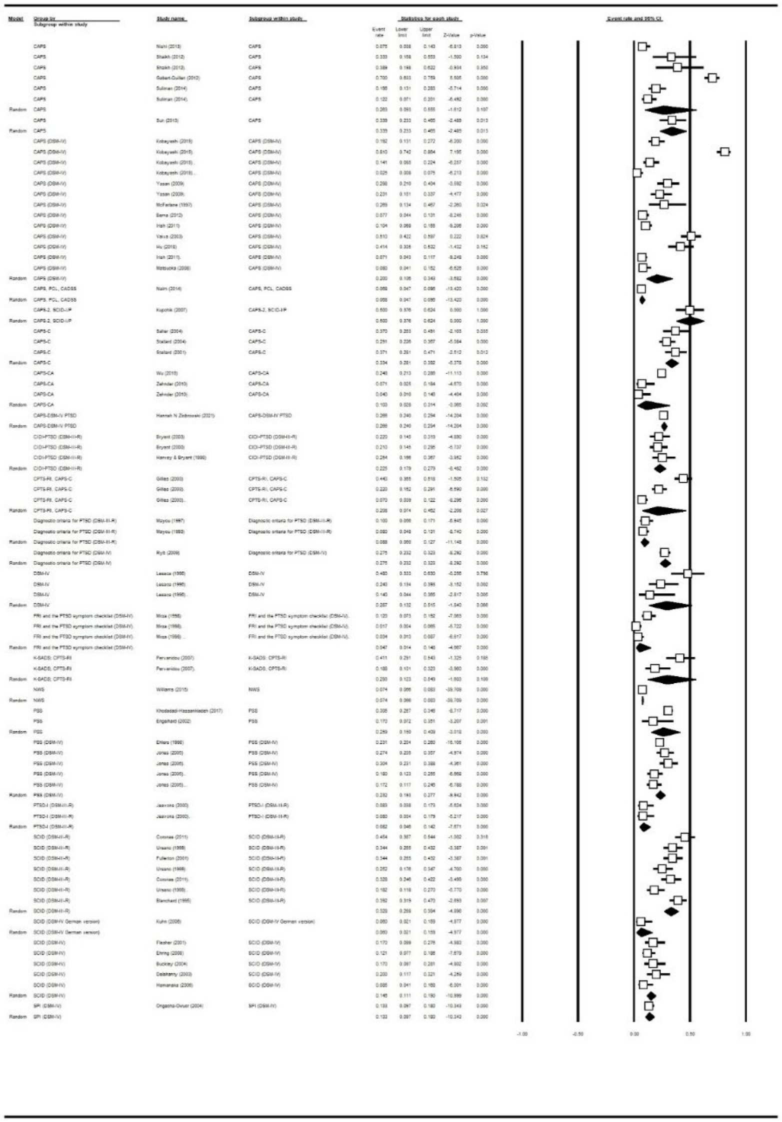

Figure S3. The forest plot of individual effect sizes within each study in Clinician-administered group based on the used checklist.

Model	Group by Subgroup within study	Study name	Subgroup within study	Event rate	Lower limit	Upper limit	Z-Value	p-Value
	Children's Revised Impact of Events Scale (CRIES-13)	Giannopoulou (2021)	Children's Revised Impact of Events Scale (CRIES-13)	0.780	0.711	0.836	6.796	0.000
Random	Children's Revised Impact of Events Scale (CRIES-13)			0.780	0.711	0.836	6.796	0.000
	IES,PSS (DSM-III-R)	Frommberger (1998)	IES,PSS (DSM-III-R)	0.184	0.130	0.254	-7.116	0.000
Random	IES,PSS (DSM-III-R)			0.184	0.130	0.254	-7.116	0.000
	IES-R	Angerpointner (2020)	IES-R	0.056	0.014	0.197	-3.897	0.000
	IES-R	Angerpointner (2020).	IES-R	0.028	0.004	0.173	-3.511	0.000
	IES-R	Papadakaki (2017)	IES-R	0.396	0.313	0.486	-2.262	0.024
	IES-R	Prakasam (2013)	IES-R	0.233	0.156	0.334	-4.671	0.000
Random	IES-R			0.165	0.067	0.351	-3.153	0.002
	Impact of Event Scale – Revised (IES-R)	Schäfer (2006)	Impact of Event Scale – Revised (IES-R)	0.250	0.162	0.365	-3.952	0.000
Random	Impact of Event Scale – Revised (IES-R)			0.250	0.162	0.365	-3.952	0.000
	Malay PostTraumatic Stress Disorder Checklist Civilian version	Bahari (2017)	Malay PostTraumatic Stress Disorder Checklist Civilian version	0.074	0.031	0.165	-5.454	0.000
Random	Malay PostTraumatic Stress Disorder Checklist Civilian version			0.074	0.031	0.165	-5.454	0.000
	PCL (DSM-IV)	Chossegros (2011)	PCL (DSM-IV)	0.180	0.150	0.215	-13.550	0.000
Random	PCL (DSM-IV)			0.180	0.150	0.215	-13.550	0.000
	PCL-5	Joormann (2022)	PCL-5	0.200	0.179	0.223	-20.040	0.000
	PCL-5	Kessler (2021)	PCL-5	0.390	0.354	0.428	-5.630	0.000
	PCL-5	Neylan (2021)	PCL-5	0.420	0.383	0.458	-4.111	0.000
	PCL-5	Yrondi (2022)	PCL-5	0.136	0.086	0.208	-7.086	0.000
	PCL-5	Yrondi (2022).	PCL-5	0.103	0.061	0.170	-7.355	0.000
Random	PCL-5			0.234	0.145	0.355	-3.946	0.000
	PCL-C	Fekadu (2019)	PCL-C	0.465	0.409	0.522	-1.209	0.227
	PCL-C	Fitzharris (2006)	PCL-C	0.029	0.004	0.177	-3.486	0.000
	PCL-C	Fitzharris (2006).	PCL-C	0.074	0.019	0.252	-3.437	0.001
	PCL-C	Fredman (2017)	PCL-C	0.421	0.334	0.513	-1.680	0.093
	PCL-C	Fredman (2017).	PCL-C	0.243	0.173	0.330	-5.204	0.000
	PCL-C	Kovacevic (2021)	PCL-C	0.355	0.292	0.424	-4.041	0.000
	PCL-C	Kovacevic (2021).	PCL-C	0.205	0.155	0.267	-7.738	0.000
	PCL-C	Ning (2017)	PCL-C	0.150	0.103	0.213	-7.980	0.000
	PCL-C	Lemos (2018)	PCL-C	0.102	0.068	0.150	-9.675	0.000
Random	PCL-C			0.219	0.141	0.325	-4.625	0.000
	PCL-S	Allenou (2010)	PCL-S	0.181	0.108	0.287	-4.932	0.000
	PCL-S	Allenou (2010).	PCL-S	0.036	0.005	0.214	-3.241	0.001
	PCL-S	Bedaso (2020)	PCL-S	0.154	0.122	0.192	-12.542	0.000
	PCL-S	LI (2021)	PCL-S	0.248	0.194	0.312	-6.876	0.000
	PCL-S	Yohannes (2018)	PCL-S	0.228	0.193	0.267	-11.350	0.000
Random	PCL-S			0.195	0.149	0.250	-8.611	0.000
	PDS (DSM-IV)	Fuglsang (2004)	PDS (DSM-IV)	0.170	0.106	0.262	-5.650	0.000
	PDS (DSM-IV)	Nightingale (2000)	PDS (DSM-IV)	0.308	0.205	0.435	-2.895	0.004
Random	PDS (DSM-IV)			0.232	0.124	0.393	-3.085	0.002
	PDS German version	Mehnert (2012) ...	PDS German version	0.080	0.030	0.196	-4.638	0.000
	PDS German version	Mehnert (2012)....	PDS German version	0.290	0.181	0.431	-2.844	0.004
Random	PDS German version			0.166	0.042	0.475	-2.089	0.037
	Penn Inventory (DSM edition not specified)	Holeva (2001)	Penn Inventory (DSM edition not specified)	0.230	0.183	0.285	-8.278	0.000
Random	Penn Inventory (DSM edition not specified)			0.230	0.183	0.285	-8.278	0.000
	PTSD-RI	Bryant (2004)	PTSD-RI	0.250	0.170	0.352	-4.412	0.000
	PTSD-RI	Bryant (2004) .	PTSD-RI	0.180	0.112	0.276	-5.402	0.000
Random	PTSD-RI			0.216	0.155	0.293	-6.192	0.000
	PTSD-RI (DSM-III-R)	Wang (2005)	PTSD-RI (DSM-III-R)	0.828	0.716	0.902	4.744	0.000
Random	PTSD-RI (DSM-III-R)			0.828	0.716	0.902	4.744	0.000
	PTSD-RI; IES	DI Gallo (1997)	PTSD-RI; IES	0.350	0.230	0.492	-2.067	0.039
Random	PTSD-RI; IES			0.350	0.230	0.492	-2.067	0.039
	RIES-C; CPTC	Meiser-Stedman (2009)	RIES-C; CPTC	0.214	0.099	0.402	-2.823	0.005
	RIES-C; CPTC	Meiser-Stedman (2009).	RIES-C; CPTC	0.107	0.035	0.284	-3.471	0.001
Random	RIES-C; CPTC			0.166	0.084	0.303	-4.048	0.000
	SRS-PTSD (DSM-III-R), IES	Smith (2007)	SRS-PTSD (DSM-III-R), IES	0.128	0.054	0.273	-4.003	0.000
Random	SRS-PTSD (DSM-III-R), IES			0.128	0.054	0.273	-4.003	0.000

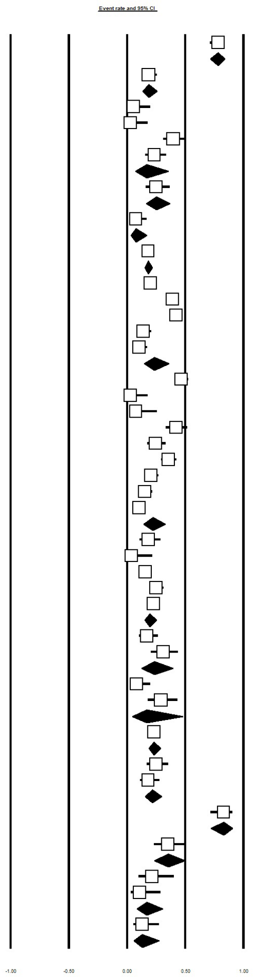

Figure S4. The forest plot of individual effect sizes within each study in self-reported group.

Model	Group by Subgroup within study	Study name	Subgroup within study	Statistics for each study				
				Event rate	Lower limit	Upper limit	Z-Value	p-Value
	1 month	Bahari (2017)	1 month	0.074	0.031	0.165	-5.454	0.000
	1 month	Bedaso (2020)	1 month	0.154	0.122	0.192	-12.542	0.000
	1 month	Fekadu (2019)	1 month	0.465	0.409	0.522	-1.209	0.227
	1 month	Fredman (2017)	1 month	0.421	0.334	0.513	-1.680	0.093
	1 month	Kovacevic (2021)	1 month	0.355	0.292	0.424	-4.041	0.000
	1 month	Yohannes (2018)	1 month	0.228	0.193	0.267	-11.350	0.000
	1 month	Mehnert (2012)....	1 month	0.290	0.181	0.431	-2.844	0.004
	1 month	Allenou (2010)	1 month	0.181	0.108	0.287	-4.932	0.000
	1 month	Allenou (2010).	1 month	0.036	0.005	0.214	-3.241	0.001
	1 month	Meiser-Stedman (2009)	1 month	0.214	0.099	0.402	-2.823	0.005
	1 month	Yrondi (2022)	1 month	0.136	0.086	0.208	-7.086	0.000
Random	1 month			0.226	0.159	0.312	-5.470	0.000
	1.5 month	Angerpointner (2020)	1.5 month	0.056	0.014	0.197	-3.897	0.000
	1.5 month	Nightingale (2000)	1.5 month	0.308	0.205	0.435	-2.895	0.004
	1.5 month	Wang (2005)	1.5 month	0.828	0.716	0.902	4.744	0.000
Random	1.5 month			0.352	0.061	0.820	-0.560	0.575
	1.5-2 months	Fitzharris (2006)	1.5-2 months	0.029	0.004	0.177	-3.486	0.000
	1.5-2 months	Fitzharris (2006).	1.5-2 months	0.074	0.019	0.252	-3.437	0.001
Random	1.5-2 months			0.054	0.017	0.154	-4.831	0.000
	2 months	Giannopoulou (2021)	2 months	0.780	0.711	0.836	6.796	0.000
	2 months	Kessler (2021)	2 months	0.390	0.354	0.428	-5.630	0.000
	2 months	Neylan (2021)	2 months	0.420	0.383	0.458	-4.111	0.000
Random	2 months			0.534	0.365	0.696	0.392	0.695
	3 months	Angerpointner (2020).	3 months	0.028	0.004	0.173	-3.511	0.000
	3 months	Ning (2017)	3 months	0.150	0.103	0.213	-7.980	0.000
	3 months	Bryant (2004)	3 months	0.250	0.170	0.352	-4.412	0.000
	3 months	Schäfer (2006)	3 months	0.250	0.162	0.365	-3.952	0.000
	3 months	Joormann (2022)	3 months	0.200	0.179	0.223	-20.040	0.000
Random	3 months			0.197	0.152	0.250	-8.912	0.000
	3-4 months	Di Gallo (1997)	3-4 months	0.350	0.230	0.492	-2.067	0.039
Random	3-4 months			0.350	0.230	0.492	-2.067	0.039
	4 months	Fredman (2017).	4 months	0.243	0.173	0.330	-5.204	0.000
	4 months	Smith (2007)	4 months	0.128	0.054	0.273	-4.003	0.000
Random	4 months			0.196	0.105	0.336	-3.778	0.000
	4-12 months	LI (2021)	4-12 months	0.248	0.194	0.312	-6.876	0.000
Random	4-12 months			0.248	0.194	0.312	-6.876	0.000
	4-6 months	Holeva (2001)	4-6 months	0.230	0.183	0.285	-8.278	0.000
Random	4-6 months			0.230	0.183	0.285	-8.278	0.000
	6 months	Chossegros (2011)	6 months	0.180	0.150	0.215	-13.550	0.000
	6 months	Frommberger (1998)	6 months	0.184	0.130	0.254	-7.116	0.000
	6 months	Kovacevic (2021).	6 months	0.205	0.155	0.267	-7.738	0.000
	6 months	Papadakaki (2017)	6 months	0.396	0.313	0.486	-2.262	0.024
	6 months	Prakasam (2013)	6 months	0.233	0.156	0.334	-4.671	0.000
	6 months	Lemos (2018)	6 months	0.102	0.068	0.150	-9.675	0.000
	6 months	Mehnert (2012) ...	6 months	0.080	0.030	0.196	-4.638	0.000
	6 months	Bryant (2004) .	6 months	0.180	0.112	0.276	-5.402	0.000
	6 months	Meiser-Stedman (2009).	6 months	0.107	0.035	0.284	-3.471	0.001
	6 months	Yrondi (2022).	6 months	0.103	0.061	0.170	-7.355	0.000
Random	6 months			0.174	0.130	0.230	-8.773	0.000
	6-8 months	Fuglsang (2004)	6-8 months	0.170	0.106	0.262	-5.650	0.000
Random	6-8 months			0.170	0.106	0.262	-5.650	0.000

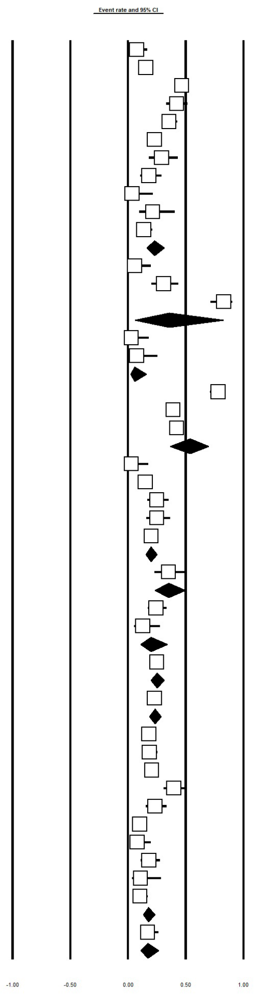

Figure S5. The forest plot for the prevalence of PTSD at different time points within self-reported group.

Model	Group by Subgroup within study	Study name	Subgroup within study	Event rate	Lower limit	Upper limit	Z-Value	p-Value
				Statistics for each study				
	Africa	Yohannes (2018)	Africa	0.228	0.193	0.267	-11.350	0.000
	Africa	Bedaso (2020)	Africa	0.154	0.122	0.192	-12.542	0.000
	Africa	Fekadu (2019)	Africa	0.465	0.409	0.522	-1.209	0.227
Fixed	Africa			0.275	0.249	0.303	-14.205	0.000
	Asia	LI (2021)	Asia	0.248	0.194	0.312	-6.876	0.000
	Asia	Ning (2017)	Asia	0.150	0.103	0.213	-7.980	0.000
	Asia	Prakasam (2013)	Asia	0.233	0.156	0.334	-4.671	0.000
	Asia	Bahari (2017)	Asia	0.074	0.031	0.165	-5.454	0.000
	Asia	Wang (2005)	Asia	0.828	0.716	0.902	4.744	0.000
Fixed	Asia			0.255	0.218	0.297	-10.095	0.000
	Europe	Kovacevic (2021)	Europe	0.355	0.292	0.424	-4.041	0.000
	Europe	Kovacevic (2021).	Europe	0.205	0.155	0.267	-7.738	0.000
	Europe	Fuglsang (2004)	Europe	0.170	0.106	0.262	-5.650	0.000
	Europe	Yrondi (2022)	Europe	0.136	0.086	0.208	-7.086	0.000
	Europe	Yrondi (2022).	Europe	0.103	0.061	0.170	-7.355	0.000
	Europe	Chossegros (2011)	Europe	0.180	0.150	0.215	-13.550	0.000
	Europe	Allenou (2010)	Europe	0.181	0.108	0.287	-4.932	0.000
	Europe	Allenou (2010).	Europe	0.036	0.005	0.214	-3.241	0.001
	Europe	Schäfer (2006)	Europe	0.250	0.162	0.365	-3.952	0.000
	Europe	Frommberger (1998)	Europe	0.184	0.130	0.254	-7.116	0.000
	Europe	Angerpointner (2020)	Europe	0.056	0.014	0.197	-3.897	0.000
	Europe	Angerpointner (2020).	Europe	0.028	0.004	0.173	-3.511	0.000
	Europe	Mehnert (2012)....	Europe	0.290	0.181	0.431	-2.844	0.004
	Europe	Mehnert (2012) ...	Europe	0.080	0.030	0.196	-4.638	0.000
	Europe	Giannopoulou (2021)	Europe	0.780	0.711	0.836	6.796	0.000
	Europe	Papadakaki (2017)	Europe	0.396	0.313	0.486	-2.262	0.024
	Europe	Lemos (2018)	Europe	0.102	0.068	0.150	-9.675	0.000
	Europe	DI Gallo (1997)	Europe	0.350	0.230	0.492	-2.067	0.039
	Europe	Nightingale (2000)	Europe	0.308	0.205	0.435	-2.895	0.004
	Europe	Meiser-Stedman (2009)	Europe	0.214	0.099	0.402	-2.823	0.005
	Europe	Meiser-Stedman (2009).	Europe	0.107	0.035	0.284	-3.471	0.001
	Europe	Smith (2007)	Europe	0.128	0.054	0.273	-4.003	0.000
	Europe	Bryant (2004)	Europe	0.250	0.170	0.352	-4.412	0.000
	Europe	Bryant (2004) .	Europe	0.180	0.112	0.276	-5.402	0.000
	Europe	Holeva (2001)	Europe	0.230	0.183	0.285	-8.278	0.000
Fixed	Europe			0.244	0.227	0.261	-23.990	0.000
	North America	Joormann (2022)	North America	0.200	0.179	0.223	-20.040	0.000
	North America	Kessler (2021)	North America	0.390	0.354	0.428	-5.630	0.000
	North America	Neylan (2021)	North America	0.420	0.383	0.458	-4.111	0.000
	North America	Fredman (2017)	North America	0.421	0.334	0.513	-1.680	0.093
	North America	Fredman (2017).	North America	0.243	0.173	0.330	-5.204	0.000
Fixed	North America			0.316	0.299	0.334	-18.528	0.000
	Oceania	Fitzharris (2006)	Oceania	0.029	0.004	0.177	-3.486	0.000
	Oceania	Fitzharris (2006).	Oceania	0.074	0.019	0.252	-3.437	0.001
Fixed	Oceania			0.054	0.017	0.154	-4.831	0.000

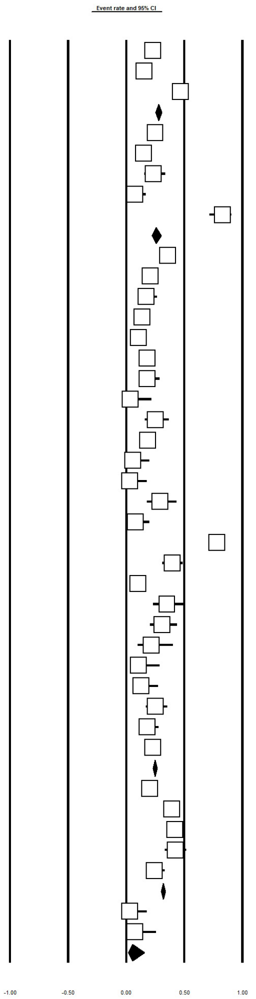

Figure S6. The forest plot for the prevalence of PTSD in different WHO regions within self-reported group.

Model	Group by Subgroup within study	Study name	Subgroup within study	Statistics for each study: Event rate	Lower limit	Upper limit	Z-Value	p-Value
	Australia	Fitzharris (2006)	Australia	0.029	0.004	0.177	-3.486	0.000
	Australia	Fitzharris (2006).	Australia	0.074	0.019	0.252	-3.437	0.001
Random	Australia			0.054	0.017	0.154	-4.831	0.000
	Balkans	Kovacevic (2021)	Balkans	0.355	0.292	0.424	-4.041	0.000
	Balkans	Kovacevic (2021).	Balkans	0.205	0.155	0.267	-7.738	0.000
Random	Balkans			0.275	0.153	0.443	-2.560	0.010
	China	LI (2021)	China	0.248	0.194	0.312	-6.876	0.000
	China	Ning (2017)	China	0.150	0.103	0.213	-7.980	0.000
Random	China			0.197	0.117	0.312	-4.500	0.000
	Denmark	Fuglsang (2004)	Denmark	0.170	0.106	0.262	-5.650	0.000
Random	Denmark			0.170	0.106	0.262	-5.650	0.000
	Ethiopia	Bedaso (2020)	Ethiopia	0.154	0.122	0.192	-12.542	0.000
	Ethiopia	Fekadu (2019)	Ethiopia	0.465	0.409	0.522	-1.209	0.227
	Ethiopia	Yohannes (2018)	Ethiopia	0.228	0.193	0.267	-11.350	0.000
Random	Ethiopia			0.265	0.130	0.466	-2.263	0.024
	France	Allenou (2010)	France	0.181	0.108	0.287	-4.932	0.000
	France	Allenou (2010).	France	0.036	0.005	0.214	-3.241	0.001
	France	Yrondi (2022)	France	0.136	0.086	0.208	-7.086	0.000
	France	Chossegros (2011)	France	0.180	0.150	0.215	-13.550	0.000
	France	Yrondi (2022).	France	0.103	0.061	0.170	-7.355	0.000
Random	France			0.147	0.110	0.194	-10.314	0.000
	Germany	Angerpointner (2020)	Germany	0.056	0.014	0.197	-3.897	0.000
	Germany	Angerpointner (2020).	Germany	0.028	0.004	0.173	-3.511	0.000
	Germany	Schäfer (2006)	Germany	0.250	0.162	0.365	-3.952	0.000
	Germany	Frommberger (1998)	Germany	0.184	0.130	0.254	-7.116	0.000
	Germany	Mehnert (2012)....	Germany	0.290	0.181	0.431	-2.844	0.004
	Germany	Mehnert (2012) ...	Germany	0.080	0.030	0.196	-4.638	0.000
Random	Germany			0.156	0.095	0.247	-5.766	0.000
	Greece	Giannopoulou (2021)	Greece	0.780	0.711	0.836	6.796	0.000
Random	Greece			0.780	0.711	0.836	6.796	0.000
	Greece, Germany and Italy	Papadakaki (2017)	Greece, Germany and Italy	0.396	0.313	0.486	-2.262	0.024
Random	Greece, Germany and Italy			0.396	0.313	0.486	-2.262	0.024
	India	Prakasam (2013)	India	0.233	0.156	0.334	-4.671	0.000
Random	India			0.233	0.156	0.334	-4.671	0.000
	Malaysia	Bahari (2017)	Malaysia	0.074	0.031	0.165	-5.454	0.000
Random	Malaysia			0.074	0.031	0.165	-5.454	0.000
	Portugal	Lemos (2018)	Portugal	0.102	0.068	0.150	-9.675	0.000
Random	Portugal			0.102	0.068	0.150	-9.675	0.000
	Scotland	DI Gallo (1997)	Scotland	0.350	0.230	0.492	-2.067	0.039
Random	Scotland			0.350	0.230	0.492	-2.067	0.039
	Taiwan	Wang (2005)	Taiwan	0.828	0.716	0.902	4.744	0.000
Random	Taiwan			0.828	0.716	0.902	4.744	0.000
	UK	Meiser-Stedman (2009)	UK	0.214	0.099	0.402	-2.823	0.005
	UK	Nightingale (2000)	UK	0.308	0.205	0.435	-2.895	0.004
	UK	Bryant (2004)	UK	0.250	0.170	0.352	-4.412	0.000
	UK	Smith (2007)	UK	0.128	0.054	0.273	-4.003	0.000
	UK	Bryant (2004) .	UK	0.180	0.112	0.276	-5.402	0.000
	UK	Meiser-Stedman (2009).	UK	0.107	0.035	0.284	-3.471	0.001
	UK	Holeva (2001)	UK	0.230	0.183	0.285	-8.278	0.000
Random	UK			0.221	0.181	0.266	-10.003	0.000
	USA	Fredman (2017)	USA	0.421	0.334	0.513	-1.680	0.093
	USA	Kessler (2021)	USA	0.390	0.354	0.428	-5.630	0.000
	USA	Neylan (2021)	USA	0.420	0.383	0.458	-4.111	0.000
	USA	Joormann (2022)	USA	0.200	0.179	0.223	-20.040	0.000
	USA	Fredman (2017).	USA	0.243	0.173	0.330	-5.204	0.000
Random	USA			0.327	0.226	0.448	-2.755	0.006

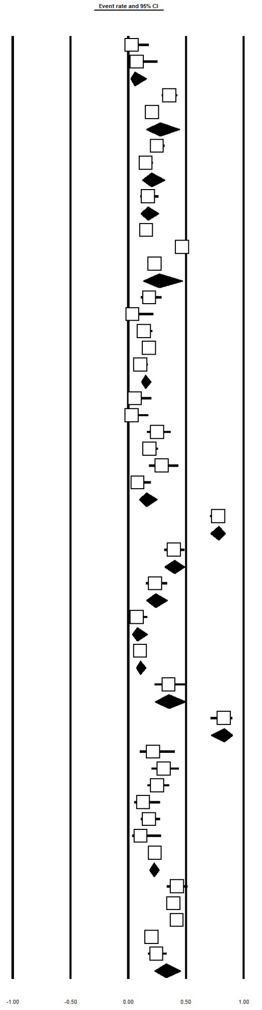

Figure S7. The forest plot for the prevalence of PTSD in different countries within self-reported group.

Model	Group by Subgroup within study	Study name	Subgroup within study	Event rate	Lower limit	Upper limit	Z-Value	p-Value
	Admitted to ICU	Papadakaki (2017)	Admitted to ICU	0.396	0.313	0.486	-2.262	0.024
Random	Admitted to ICU			0.396	0.313	0.486	-2.262	0.024
	Attended ED	Schäfer (2006)	Attended ED	0.250	0.162	0.365	-3.952	0.000
	Attended ED	Fuglsang (2004)	Attended ED	0.170	0.106	0.262	-5.650	0.000
	Attended ED	Meiser-Stedman (2009)	Attended ED	0.214	0.099	0.402	-2.823	0.005
	Attended ED	Meiser-Stedman (2009).	Attended ED	0.107	0.035	0.284	-3.471	0.001
Random	Attended ED			0.198	0.149	0.259	-7.938	0.000
	hospitalized	Fitzharris (2006)	hospitalized	0.029	0.004	0.177	-3.486	0.000
	hospitalized	Fitzharris (2006).	hospitalized	0.074	0.019	0.252	-3.437	0.001
	hospitalized	Chossegros (2011)	hospitalized	0.180	0.150	0.215	-13.550	0.000
	hospitalized	Bedaso (2020)	hospitalized	0.154	0.122	0.192	-12.542	0.000
	hospitalized	Wang (2005)	hospitalized	0.828	0.716	0.902	4.744	0.000
Random	hospitalized			0.215	0.090	0.431	-2.493	0.013
	Hospitalized minimum of bone fracture	Frommberger (1998)	Hospitalized minimum of bone fracture	0.184	0.130	0.254	-7.116	0.000
Random	Hospitalized minimum of bone fracture			0.184	0.130	0.254	-7.116	0.000
	Mild	DI Gallo (1997)	Mild	0.350	0.230	0.492	-2.067	0.039
	Mild	Angerpointner (2020)	Mild	0.056	0.014	0.197	-3.897	0.000
	Mild	Angerpointner (2020).	Mild	0.028	0.004	0.173	-3.511	0.000
	Mild	Smith (2007)	Mild	0.128	0.054	0.273	-4.003	0.000
	Mild	Fekadu (2019)	Mild	0.465	0.409	0.522	-1.209	0.227
Random	Mild			0.179	0.074	0.374	-2.964	0.003
	Mild, Moderate, Serious, Critical	Kovacevic (2021)	Mild, Moderate, Serious, Critical	0.355	0.292	0.424	-4.041	0.000
	Mild, Moderate, Serious, Critical	Kovacevic (2021).	Mild, Moderate, Serious, Critical	0.205	0.155	0.267	-7.738	0.000
	Mild, Moderate, Serious, Critical	LI (2021)	Mild, Moderate, Serious, Critical	0.248	0.194	0.312	-6.876	0.000
	Mild, Moderate, Serious, Critical	Bahari (2017)	Mild, Moderate, Serious, Critical	0.074	0.031	0.165	-5.454	0.000
Random	Mild, Moderate, Serious, Critical			0.221	0.143	0.325	-4.656	0.000
	Minor, or hospitalized	Bryant (2004)	Minor, or hospitalized	0.250	0.170	0.352	-4.412	0.000
	Minor, or hospitalized	Bryant (2004) .	Minor, or hospitalized	0.180	0.112	0.276	-5.402	0.000
Random	Minor, or hospitalized			0.216	0.155	0.293	-6.192	0.000
	Moderate	Mehnert (2012)....	Moderate	0.290	0.181	0.431	-2.844	0.004
Random	Moderate			0.290	0.181	0.431	-2.844	0.004
	Moderate, Serious	Prakasam (2013)	Moderate, Serious	0.233	0.156	0.334	-4.671	0.000
Random	Moderate, Serious			0.233	0.156	0.334	-4.671	0.000
	NM	Giannopoulou (2021)	NM	0.780	0.711	0.836	6.796	0.000
	NM	Joormann (2022)	NM	0.200	0.179	0.223	-20.040	0.000
	NM	Kessler (2021)	NM	0.390	0.354	0.428	-5.630	0.000
	NM	Neylan (2021)	NM	0.420	0.383	0.458	-4.111	0.000
	NM	Yrondi (2022)	NM	0.136	0.086	0.208	-7.086	0.000
	NM	Yrondi (2022).	NM	0.103	0.061	0.170	-7.355	0.000
	NM	Ning (2017)	NM	0.150	0.103	0.213	-7.980	0.000
	NM	Lemos (2018)	NM	0.102	0.068	0.150	-9.675	0.000
	NM	Yohannes (2018)	NM	0.228	0.193	0.267	-11.350	0.000
	NM	Nightingale (2000)	NM	0.308	0.205	0.435	-2.895	0.004
	NM	Allenou (2010)	NM	0.181	0.108	0.287	-4.932	0.000
	NM	Allenou (2010).	NM	0.036	0.005	0.214	-3.241	0.001
Random	NM			0.235	0.162	0.329	-4.962	0.000
	Serious	Holeva (2001)	Serious	0.230	0.183	0.285	-8.278	0.000
	Serious	Fredman (2017)	Serious	0.421	0.334	0.513	-1.680	0.093
	Serious	Fredman (2017).	Serious	0.243	0.173	0.330	-5.204	0.000
	Serious	Mehnert (2012) ...	Serious	0.080	0.030	0.196	-4.638	0.000
Random	Serious			0.242	0.148	0.369	-3.707	0.000

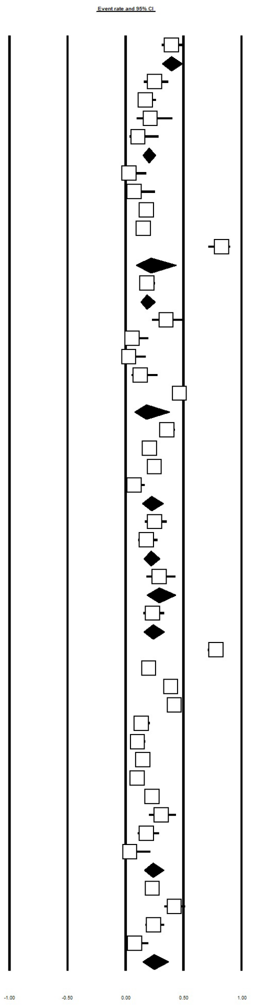

Figure S8. The forest plot for the prevalence of PTSD based on injury severity within self-reported group.

Model	Group by Subgroup within study	Study name	Subgroup within study	Statistics for each study				
				Event rate	Lower limit	Upper limit	Z-Value	p-Value
	Adult	Angerpointner (2020)	Adult	0.056	0.014	0.197	-3.897	0.000
	Adult	Angerpointner (2020).	Adult	0.028	0.004	0.173	-3.511	0.000
	Adult	Bahari (2017)	Adult	0.074	0.031	0.165	-5.454	0.000
	Adult	Bedaso (2020)	Adult	0.154	0.122	0.192	-12.542	0.000
	Adult	Chossegros (2011)	Adult	0.180	0.150	0.215	-13.550	0.000
	Adult	Fekadu (2019)	Adult	0.465	0.409	0.522	-1.209	0.227
	Adult	Fitzharris (2006)	Adult	0.029	0.004	0.177	-3.486	0.000
	Adult	Fitzharris (2006).	Adult	0.074	0.019	0.252	-3.437	0.001
	Adult	Fredman (2017)	Adult	0.421	0.334	0.513	-1.680	0.093
	Adult	Fredman (2017).	Adult	0.243	0.173	0.330	-5.204	0.000
	Adult	Frommberger (1998)	Adult	0.184	0.130	0.254	-7.116	0.000
	Adult	Fuglsang (2004)	Adult	0.170	0.106	0.262	-5.650	0.000
	Adult	Giannopoulou (2021)	Adult	0.780	0.711	0.836	6.796	0.000
	Adult	Holeva (2001)	Adult	0.230	0.183	0.285	-8.278	0.000
	Adult	Joormann (2022)	Adult	0.200	0.179	0.223	-20.040	0.000
	Adult	Kessler (2021)	Adult	0.390	0.354	0.428	-5.630	0.000
	Adult	Kovacevic (2021)	Adult	0.355	0.292	0.424	-4.041	0.000
	Adult	Kovacevic (2021).	Adult	0.205	0.155	0.267	-7.738	0.000
	Adult	Lemos (2018)	Adult	0.102	0.068	0.150	-9.675	0.000
	Adult	LI (2021)	Adult	0.248	0.194	0.312	-6.876	0.000
	Adult	Mehnert (2012) ...	Adult	0.080	0.030	0.196	-4.638	0.000
	Adult	Mehnert (2012)....	Adult	0.290	0.181	0.431	-2.844	0.004
	Adult	Neylan (2021)	Adult	0.420	0.383	0.458	-4.111	0.000
	Adult	Nightingale (2000)	Adult	0.308	0.205	0.435	-2.895	0.004
	Adult	Ning (2017)	Adult	0.150	0.103	0.213	-7.980	0.000
	Adult	Papadakaki (2017)	Adult	0.396	0.313	0.486	-2.262	0.024
	Adult	Prakasam (2013)	Adult	0.233	0.156	0.334	-4.671	0.000
	Adult	Smith (2007)	Adult	0.128	0.054	0.273	-4.003	0.000
	Adult	Wang (2005)	Adult	0.828	0.716	0.902	4.744	0.000
	Adult	Yohannes (2018)	Adult	0.228	0.193	0.267	-11.350	0.000
	Adult	Yrondi (2022)	Adult	0.136	0.086	0.208	-7.086	0.000
	Adult	Yrondi (2022).	Adult	0.103	0.061	0.170	-7.355	0.000
Random	Adult			0.234	0.189	0.286	-8.618	0.000
	Children	Allenou (2010)	Children	0.181	0.108	0.287	-4.932	0.000
	Children	Allenou (2010).	Children	0.036	0.005	0.214	-3.241	0.001
	Children	Bryant (2004)	Children	0.250	0.170	0.352	-4.412	0.000
	Children	Bryant (2004) .	Children	0.180	0.112	0.276	-5.402	0.000
	Children	DI Gallo (1997)	Children	0.350	0.230	0.492	-2.067	0.039
	Children	Meiser-Stedman (2009)	Children	0.214	0.099	0.402	-2.823	0.005
	Children	Meiser-Stedman (2009).	Children	0.107	0.035	0.284	-3.471	0.001
	Children	Schäfer (2006)	Children	0.250	0.162	0.365	-3.952	0.000
Random	Children			0.215	0.164	0.277	-7.611	0.000

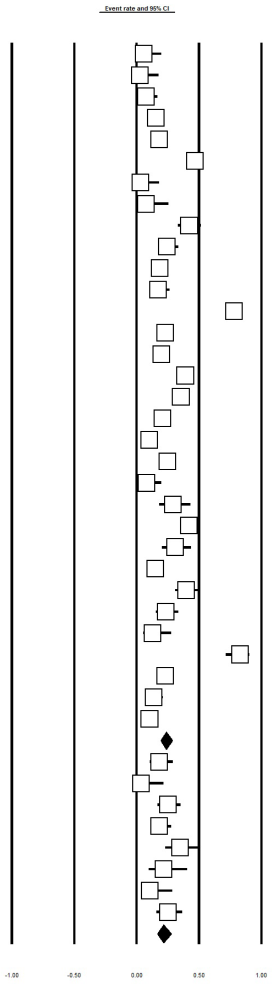

Figure S9. The forest plot for the prevalence of PTSD based on age within self-reported group.

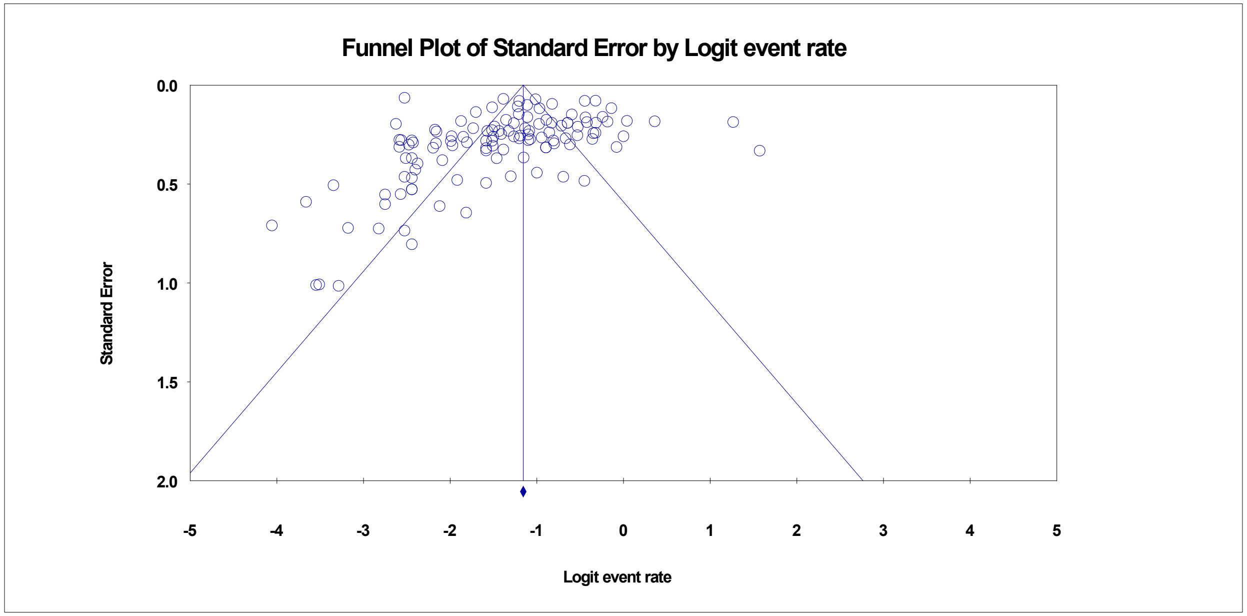

Figure S10. Funnel plot of publication bias.

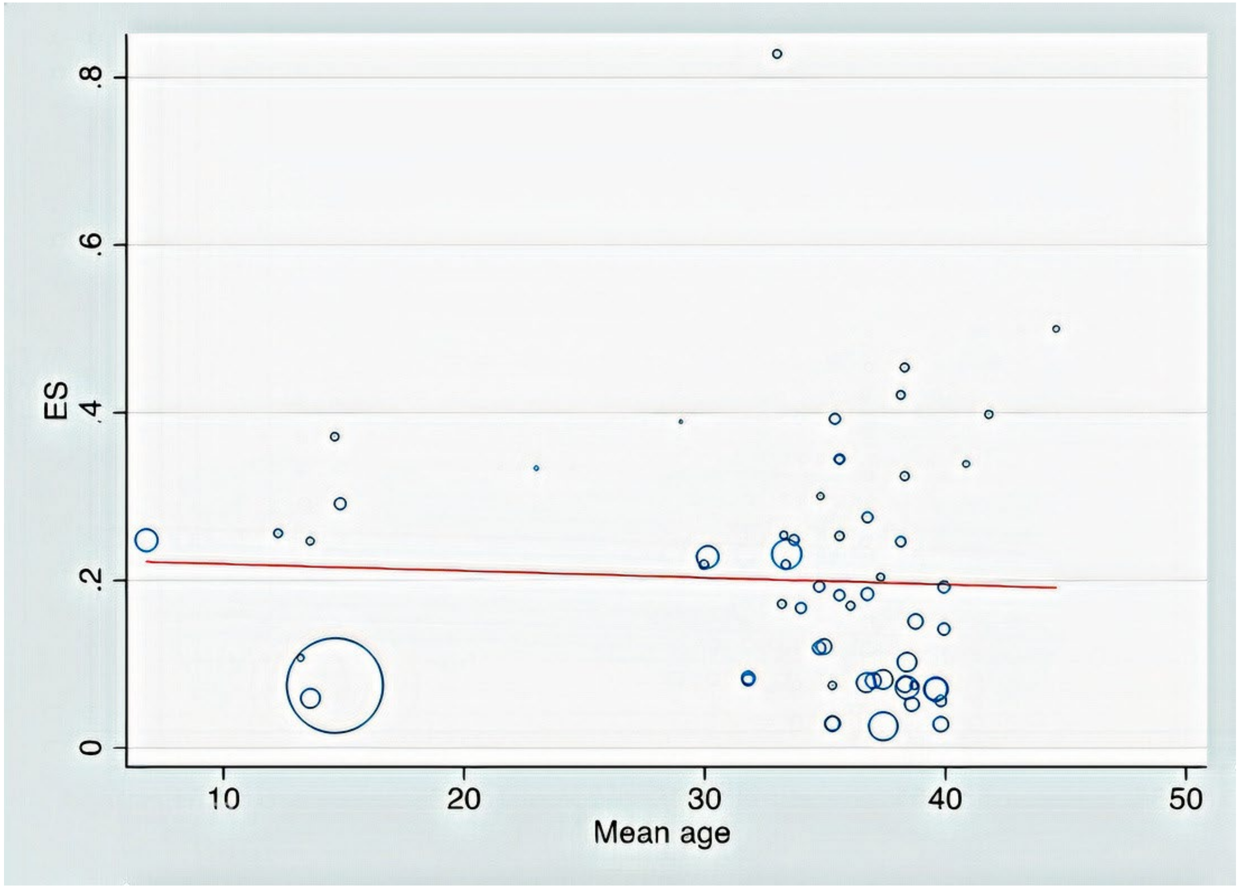

Figure S11. The results of meta-regression.
